# 4CzIPN Photosensitizes the Cobaloxime‐Catalyzed Light‐Driven Hydrogen Evolution Reaction

**DOI:** 10.1002/cssc.70942

**Published:** 2026-08-22

**Authors:** Alexander Tombrink, Benedikt Callies, Stephan Kupfer, Daniel Kowalcyk, Benedikt Wiedemann, Dirk Ziegenbalg, Kalina Peneva, Birgit Esser

**Affiliations:** ^1^ Institute of Organic Chemistry II and Advanced Materials Ulm University Ulm Germany; ^2^ Institute of Organic Chemistry and Macromolecular Chemistry Friedrich Schiller University Jena Jena Germany; ^3^ Institute of Physical Chemistry Friedrich Schiller University Jena Jena Germany; ^4^ Institute of Chemical Engineering Ulm University Ulm Germany; ^5^ Center for Energy and Environmental Chemistry Jena (CEEC Jena) Jena Germany; ^6^ Jena Center for Soft Matter (JCSM) Friedrich Schiller University Jena Jena Germany; ^7^ CELEST Green Energy Lab Ulm Ulm University Ulm Germany; ^8^ Electrochemical Energy Storage Helmholtz Institute Ulm (HIU) Ulm Germany

**Keywords:** excited‐state quenching, noble‐metal‐free, photocatalysis, photosensitizer, reaction rate constant, thermally activated delayed fluorescence, triplet state

## Abstract

Thermally activated delayed fluorescence (TADF) compounds have found great application as photocatalysts in recent years. Their use as photosensitizers (PS), however, is still limited although they have huge potential as abundant alternatives to traditional noble metal‐based PSs, such as [Ru(bpy)_3_]^2+^. We herein investigate the TADF compound 2,4,5,6‐tetra(9*H*‐carbazol‐9‐yl)isophthalonitrile (4CzIPN) as a PS together with the catalyst cobaloxime (Co(dmgH)_2_(py)Cl) in the photochemical hydrogen evolution reaction (HER). Systematic optimization of the solvent composition, 4CzIPN and catalyst concentration, and sacrificial electron donor (SED) identified ammonium ascorbate as the most effective SED under the conditions tested. Under optimized conditions, the system reached a TON of 6451 ± 82 after 20 h irradiation. Stern–Volmer quenching experiments and scalar‐relativistic quantum‐chemical calculations provided rate constants for the different processes in the photosensitization mechanism, demonstrating that the quenching of the excited singlet state (S_1_) of 4CzIPN by ammonium ascorbate is by far more dominant than the quenching of its excited triplet state (T_1_). This work demonstrates that 4CzIPN can sensitize the cobaloxime‐catalyzed light‐driven HER with ammonium ascorbate as the SED and provides mechanistic insight into the role of singlet‐ and triplet‐state quenching in TADF photosensitization.

## Graphical abstract

2,4,5,6‐Tetra(9*H*‐carbazol‐9‐yl)isophthalonitrile (4CzIPN) as photosensitizer enables turnover numbers above 5000 in the cobaloxime‐catalyzed hydrogen evolution reaction via efficient quenching of its excited singlet state by ascorbate.

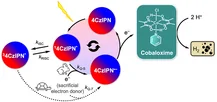

## 1 Introduction

Climate change necessitates transitioning toward green and renewable energy. While solar and wind energy are abundantly available, the capabilities for their temporary storage are still limited. One approach is to convert light into chemical bond energy, e.g., into solar fuels such as molecular hydrogen (H_2_). Hydrogen can be produced from water and can then later be converted into electricity using fuel cells, whereby the combustion product is again water. Light‐driven catalysis can enable such hydrogen production from (sun)light energy. Focusing on the half‐reaction of proton reduction (hydrogen production), there are numerous known catalysts that can drive this light‐driven hydrogen evolution reaction (HER), such as thiomolybdates [1], noble metals [2], metal oxides [3], and metal‐organic frameworks [4]. Among these, the cobalt complex Co(dmgH)_2_(py)Cl (cobaloxime) stands out, as it contains no expensive noble metals and was designed as a model compound to mimic nature’s Vitamin B12 [5]. Co(dmgH)_2_(py)Cl (cobaloxime) was first applied as a molecular HER catalyst in 1983 in combination with [Ru(bpy)_3_]^2+^ as the photosensitizer (PS) and triethanolamine (TEOA) as the sacrificial electron donor (SED) [6]. Since then, cobaloxime‐based HER systems have been combined with a range of metal‐containing and organic PSs. For example, Ru‐, Ir‐, and Pt‐based PSs have afforded turnover number (TON) values between 91 after 1 h and 789 after 24 h under optimized conditions [5]. Noble‐metal‐free PSs have also shown promising performance, including a selenium‐substituted rhodamine dye with a reported TON of 9000 after 8 h and ketocoumarin PSs with TONs of up to 3955 after 20 h [7, 8]. Ming and coworkers reported a particularly high TON of 780,000 (after 160 h) using an anthraquinone dye and 1,3‐dimethyl‐2‐phenyl‐2,3‐dihydro‐1H‐benzo[d]imidazole (BIH) as the SED [9]. However, these values should not be directly ranked because the reported systems substantially differ in solvent composition, SED, irradiation time, catalyst and PS loading, and in whether TON is calculated with respect to the catalyst or the PS. In recent years, a new class of organic dyes for photocatalysis has emerged: thermally activated delayed fluorescence (TADF) compounds possess pronounced visible‐light absorption and long‐lived excited (triplet) states in combination with redox potentials comparable to those of noble‐metal based PSs [10, 11, 12]. They are usually designed in a donor–acceptor fashion with a significant dihedral angle between the donor and the acceptor part of the molecule. This results in a spatial separation between the HOMO and LUMO and a small energy gap between the first excited singlet and triplet states (Δ*E*_ST_ < 0.2 eV). It is for this reason that the intersystem crossing (ISC) is followed by a reverse ISC (RISC), and thus delayed fluorescence may occur [10, 13]. Initially developed as emitters for organic light‐emitting diodes (OLEDs), TADF compounds are now well established as organic photoredox catalysts [10, 11, 12], however, their use as PS is still a rather new concept. In only one example, they were used in HER [14], where Yu and coworkers reported a TON of 2023 (60 h) with 2,4,5,6‐tetra(9*H*‐carbazol‐9‍‐‍yl)isophthalonitrile (4CzIPN) as TADF PS, PdCl_2_(PPh_3_)_2_ as catalyst, and triethylamine (TEA) as SED [14]. 4CzIPN also showed good performance as a PS in CO_2_ reduction [15], and is also widely used as photoredox catalyst in organic transformations [16].

The SED is also an important component in light‐driven HER. As it is used in high amounts, sustainable and nontoxic compounds are desirable. Ascorbic acid (Vitamin C, HAsc) is a good choice and has long been known as an antioxidant and as natural preservation agent [17]. After deprotonation (p*K*_a_ = 4.17), ascorbate (Asc^−^) is obtained, which is reducing and can act as an SED. Its good solubility in water allows light‐driven HER to be performed in an aqueous solution at neutral or slightly acidic pH values. This renders ascorbic acid and its salt desirable as an eco‐friendly SED for proton reduction, as shown on many occasions [7, 18, 19].

**FIGURE 1:**
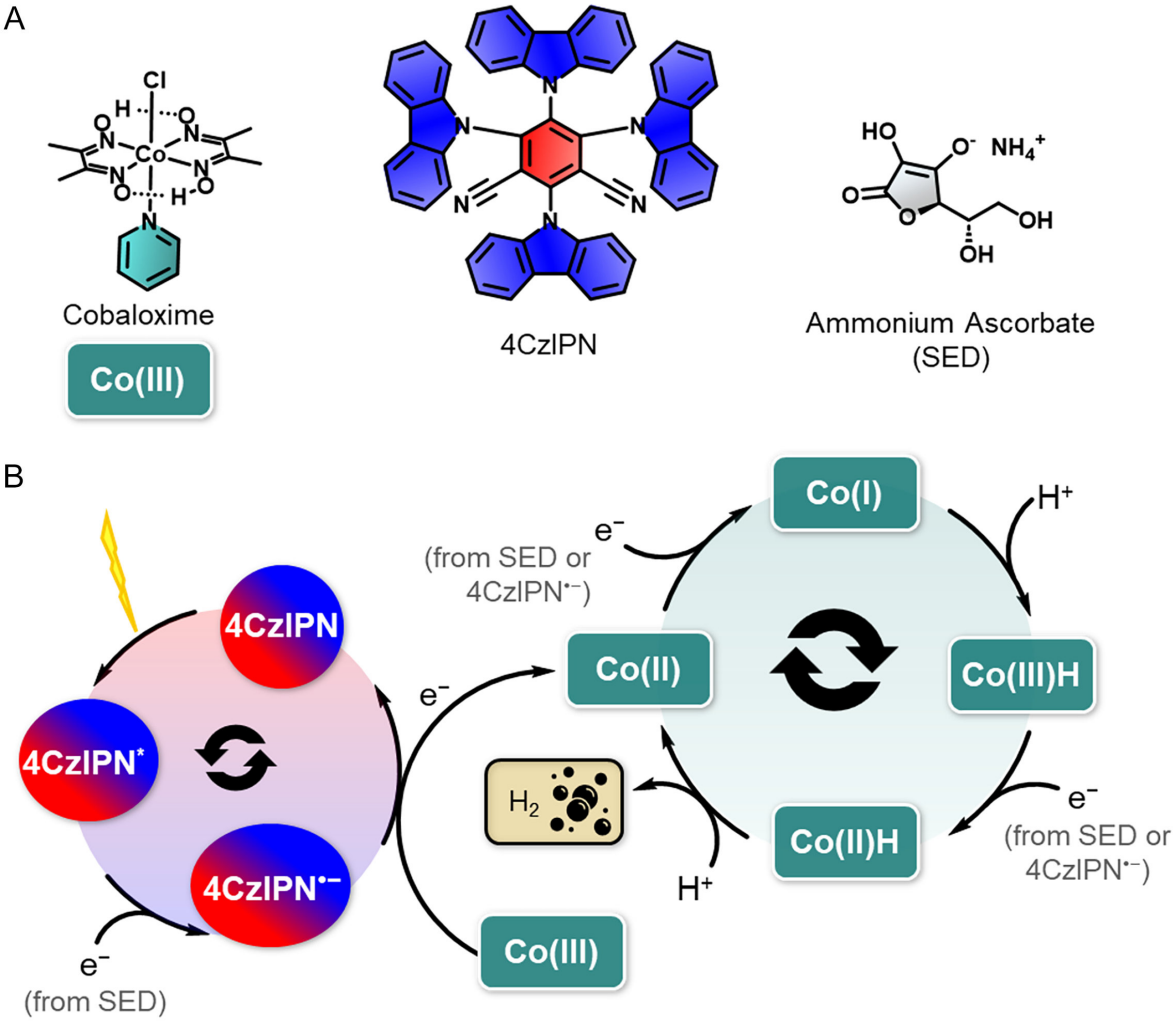
(A) Structure of the herein used HER catalyst Co(dmgH)_2_(py)Cl (cobaloxime), the PS 2,4,5,6‐tetra(9*H*‐carbazol‐9‐yl)isophthalonitrile (4CzIPN), and the SED ammonium ascorbate. (B) Proposed catalytic cycle for the hydrogen‐evolution reaction via reduction of the cobaloxime catalyst by the 4CzIPN radical anion [14, 20].

Herein, we investigate a three‐component system for HER, comprising the bio‐inspired catalyst cobaloxime, the organic PS 4CzIPN, and ammonium ascorbate as the SED (Figure 1). Optimal catalytic conditions were identified using a multibatch screening photoreactor developed by Ziegenbalg and coworkers [21], by varying the water content of an acetonitrile/water solvent mixture and changing the concentration of the PS. Stern–Volmer quenching experiments and scalar‐relativistic (SR) quantum chemical calculations provide insights with respect to the underlying mechanism.

## 2 Results and Discussion

### 2.1 Electronic and Photophysical Properties

**FIGURE 2:**
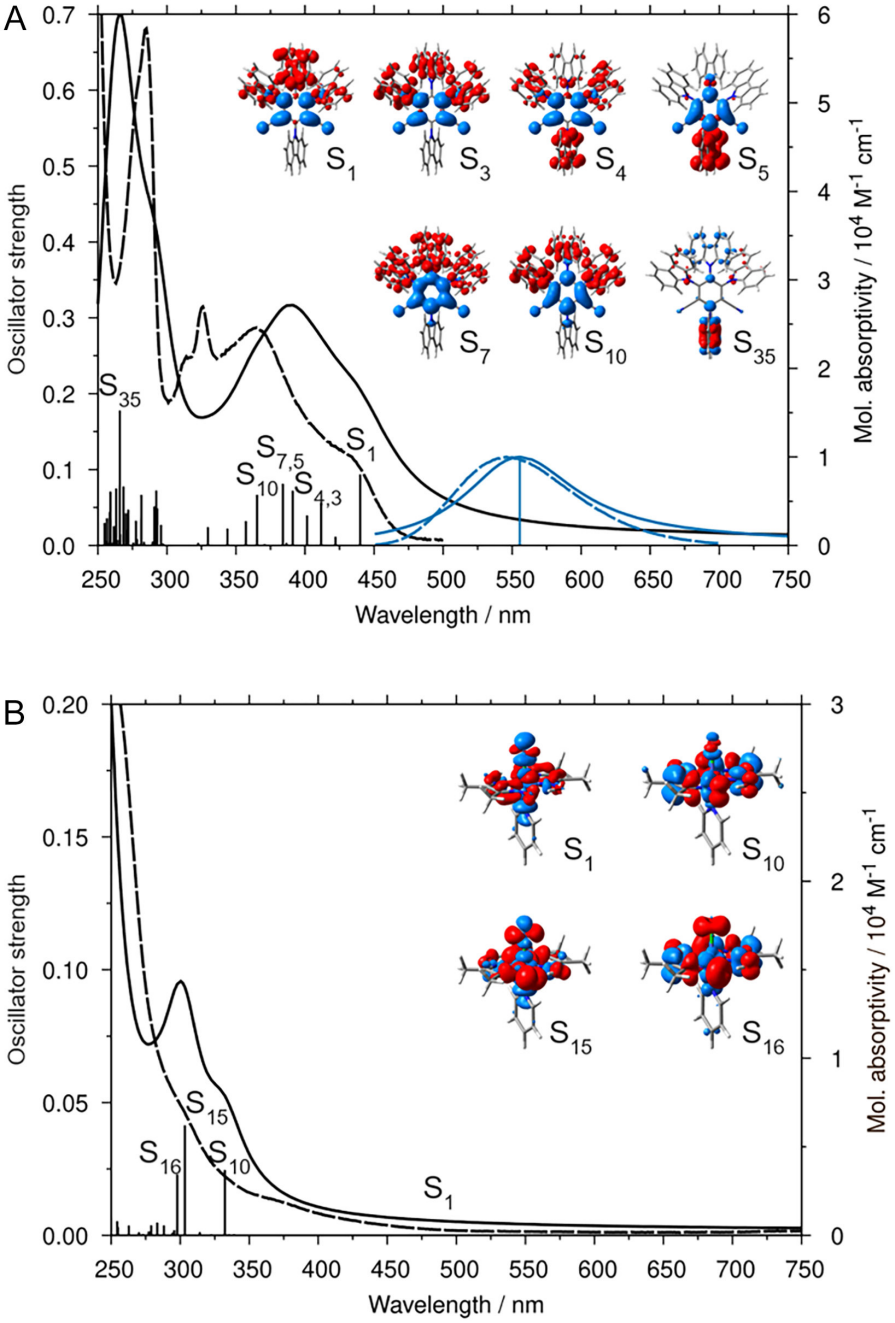
(A) Experimental (dashed black) and simulated (solid black) electronic absorption spectra of 4CzIPN in acetonitrile. Prominent transitions are labeled and their electronic nature is visualized by means of charge‐density‐difference plots (CDDs; charge‐transfer occurs from red to blue). Measured and calculated (from relaxed S_1_) emission spectra are shown in blue (dashed and solid, respectively). (B) Experimental and TDDFT‐predicted electronic absorption spectra of cobaloxime in acetonitrile, with the lowest energy dipole‐forbidden metal‐centered transition as well as dipole‐allowed transitions indicated. For the determination of molar absorptivity, 4CzIPN was measured at concentrations of 2.79 · 10−5, 2.79 · 10−6, 1.39 · 10−6, and 6.97 · 10−7 M and cobaloxime was measured at concentrations of 2.33 · 10−5, 1.16 · 10−5, 5.82 · 10−6, and 2.91 · 10−6 M.

We first investigated the electronic and photophysical properties of 4CzIPN as PS and cobaloxime as catalyst, which are of high relevance for the light‐driven HER. The UV/Vis absorption spectrum of 4CzIPN in acetonitrile (Figure 2A) features a broad and structured absorption band extending from roughly 475–300 nm with a molar absorptivity of 24,615 L mol^−1 ^cm^−1^ at 365 nm. In agreement with recent computational and spectroscopic–theoretical studies addressing the photophysical properties of 4CzIPN and its derivatives [22, 23, 24, 25], our (scalar relativistic (SR)) time‐dependent density functional theory simulations (TDDFT at the PBE0/def2svp [26, 27] level of theory; see Supporting Information for computational details) reveal several dipole‐allowed charge‐transfer (CT) transitions from the π orbitals of the carbazol donor groups to the isophthalonitrile moiety. In particular, the computational focus was set on elucidating the nature of the electronic transitions underlying the main absorption features between 475 and 300 nm as well as at roughly 365 nm (in acetonitrile), which associate this broad band with transitions into, e.g., S_1_, S_3_, S_4_, S_5_, S_7_, and S_10_ states at 440, 412, 402, 391, 384, and 365 nm (see Figure 2A and Table S1). At higher excitation energies, local excitations, such as S_35_ (266 nm), contribute to the measured absorption feature at 285 nm.

In contrast to 4CzIPN, the cobaloxime catalyst does not show electronic absorption within the visible region (Figure 2B). Noteworthy, the electronic transitions in this spectral range, e.g., into the S_1_ and S_2_ metal‐centered (MC) states at 543 and 486 nm, are dipole forbidden. These energetically low‐lying MC states are not directly involved in the initial photophysics. However, such MC states featuring a singly populated $d_{z^{2}}$ orbital in a simplistic ligand‐field picture, or rather a$\sigma_{z^{2}}^{*}$ orbital in a molecular orbital approach, will lead to (partial) dissociation of the axial halide (see Computational section in the Supporting Information) [28, 29, 30], which is key for the subsequently investigated catalytic process. Finally, weakly dipole‐allowed π–π* transitions of the equatorial ligand into, i.e., S_10_, S_15_, and S_16_, are predicted at 332, 303, and 298 nm (Table S6), respectively, contributing to the absorption at roughly 300 nm.

Time‐resolved emission spectroscopy of 4CzIPN in an aqueous MeCN solution shows an expected biexponential decay (see Figure S1) with a lifetime of *τ*_PF_ = 6 ns for the prompt fluorescence (PF) and a lifetime of *τ*_DF_ = 1522 ns for the delayed fluorescence (DF). This is in accordance with the two emissive components reported in the literature [31]. This feature is expected to be beneficial for photosensitization since a longer excited‐state lifetime would increase the probability of collision with an SED molecule.

Cyclic voltammograms of 4CzIPN and cobaloxime were recorded in MeCN to determine the redox potentials under conditions comparable to those used for the light‐driven HER experiments (Figure S2). The measured values agree well with literature data [32, 33, 34, 35]. With *E*_1/2_ = −1.61 V versus Fc/Fc^+^ for the redox couple 4CzIPN/4CzIPN^**•**−^, the reduction potential of 4CzIPN is sufficiently negative to reduce the cobaloxime catalyst from Co(III) to Co(II) and from Co(II) to Co(I), for which potentials of *E*_1/2_(Co^3+^/Co^2+^) = −0.95 V and *E*_1/2_(Co^2+^/Co^+^) = −1.51 V versus Fc/Fc^+^ were determined, respectively. The excited‐state redox potential of 4CzIPN with E*_1/2_(4CzIPN*/4CzIPN^**•**−^) = 1.12 V is substantially more positive than the redox potential of ascorbate *E*_ox_(HA^−^/HA^•^) = 0.12 V [17] versus Fc/Fc^+^. These data support a thermodynamically favorable reductive quenching of excited 4CzIPN* by ascorbate, followed by reduction of the cobaloxime catalyst by the resulting 4CzIPN radical anion [7, 32, 36]. In addition, UV/Vis spectroelectrochemistry (SEC) of 4CzIPN in acetonitrile was performed (Figure S3). Upon applying a potential of −1.45 V, the radical anion 4CzIPN^**•**−^ was generated and showed a characteristic absorption band at 471 nm.

In the case of the cobaloxime catalyst, further density functional theory (DFT) calculations were performed in order to elucidate the electronic character of the redox and spin‐state intermediates associated with the first and second reduction event during HER catalysis (Figure 1B). For the Co complex, the structure of the nonreduced singlet Co(III) species was optimized using DFT, while the singly reduced cobalt complex Co(II) was investigated in doublet (D_0_) and quartet multiplicity (Q_0_). These calculations reveal that formation of the respective doublet species is thermodynamically favored (see Table S7). Single reduction leads to a partial occupation of the $d_{z^{2}}$ orbital or the $\sigma_{z^{2}}^{*}$ orbital in a molecular orbital picture (Figure 2B), resulting in the formation of a Co(II) low‐spin intermediate in d^7^ configuration. This alternation of the electronic structure leads to a significant elongation of the axial Co─Cl and Co─py bond lengths from 2.24 and 1.95 Å (S_0_ geometry) to 2.54 and 2.29 Å, respectively, within the fully optimized singly reduced doublet redox species. The energetically less stable (by 1.38 eV or 133 kJ mol^−1^), see Table S7, singly reduced quartet delocalizes the three unpaired orbitals over the metal center, and the lowest‐energy π* orbital of the (pseudo‐)square‐planar equatorial ligand. Thus, such species reflect a singly reduced ligand sphere in combination with a high‐spin (triplet) Co(III). Upon the second reduction event, dissociation of the chloride ligand is observed within the thermodynamically preferred singlet species, due to the doubly occupied $\sigma_{z^{2}}^{*}$ orbital. This pyramidal Co(I) complex features a d^8^ configuration (see Table S7 for details). Such partial loss of the axial halide ligand is typical for cobaloxime‐based catalysts [28, 29, 30].

### 2.2 Light‐Driven Hydrogen Evolution

Light‐driven HER was investigated using 4CzIPN as PS and cobaloxime [Co(dmgH)2(py)Cl] as the catalyst in a multibatch screening photoreactor, equipped with two LED modules, each comprising two LEDs (*λ *  =  453 nm) (Table S8) [21]. Hydrogen evolution was quantified by gas chromatography (see Supporting Information for details). Dimethylglyoxime (dmgH_2_) was added to stabilize the cobaloxime catalyst under photocatalytic conditions. Cobaloxime complexes are known to undergo ligand dissociation and structural changes during catalysis, particularly under reductive turnover, and the ligand environment plays a key role in determining catalyst stability and activity [37].

As an initial solvent test, the catalytic system was examined in MeOH/H_2_O 4:1 v/v using ammonium ascorbate as the SED. Under these conditions, only a low hydrogen evolution was observed, corresponding to a TON of 17 ± 3.5 after 20 h. We attribute this low activity mainly to the limited solubility of 4CzIPN in this solvent mixture. Replacing MeOH with MeCN led to a pronounced increase in activity already under nonoptimized conditions, giving a TON of 1358 ± 165 after 20 h. Based on this improvement, subsequent optimization experiments were carried out in MeCN/H_2_O mixtures (for a detailed description of the preparation of the catalytic mixtures see Tables S9–S12).

The optimization focused on two parameters: the water content of the MeCN/H_2_O mixture and the concentration of 4CzIPN. The water content was varied between 15 and 40 vol.% H_2_O, while the 4CzIPN concentration was compared at 1 and 0.1 mM. In addition, one series of experiments was performed in the presence of PDha‐g‐PEG to evaluate whether a polymeric matrix affects the catalytic performance. The full screening data are summarized in Table 1.

**TABLE 1:** Screening of the MeCN/H_2_O ratio and 4CzIPN concentration for light‐driven HER. TONs were determined after 20 h irradiation at 453 nm using 2 *µ*M cobaloxime, 1 mg/mL dmgH_2_, and 0.1 M ammonium ascorbate.

H_2_O content in MeCN/H_2_O	1 mM 4CzIPN	0.1 mM 4CzIPN	0.1 mM 4CzIPN + 1 mg/mL PDha‐g‐PEG
15 vol.%	2720 ± 102	4190 ± 245	1910 ± 105
20 vol.%	**3960 ± 119**	**5030 ± 166**	2170 ± 702
30 vol.%	3590 ± 139	4650 ± 83	**3320 ± 127**
40 vol.%	2820 ± 102	3250 ± 398	2080 ± 159

*Note:* TON values were calculated with respect to the catalyst. Values are reported as mean ± standard deviation from triplicate measurements. Highest values are highlighted in bold.

At a 4CzIPN concentration of 1 mM, the highest activity was obtained at 20 vol.% H_2_O, with a TON of 3960 ± 119 after 20 h. Lowering the 4CzIPN concentration to 0.1 mM further improved the catalytic performance, giving the highest TON of 5030 ± 166 under otherwise identical conditions. This increase suggests that the higher dye concentration does not benefit the reaction, most likely because of concentration‐dependent deactivation processes such as self‐quenching, inner‐filter effects, or reduced light penetration through the reaction solution.

Previous studies indicated that the addition of the polyampholytic graft copolymer poly(dehydroalanine)‐graft‐poly(ethylene glycol) (PDha‐g‐PEG) can improve HER activity by increasing the apparent solubility or dispersion of poorly soluble PSs [38, 39, 40, 41]. We therefore tested PDha‐g‐PEG in the present system, since partial precipitation was observed in samples with higher water content before irradiation. However, under the conditions tested, the addition of PDha‐g‐PEG did not improve the catalytic performance. The highest TON obtained in the presence of PDha‐g‐PEG was 3320 ± 127 at 30 vol.% H_2_O, which remained lower than the best value obtained without the polymer additive. These results suggest that improved dispersion of 4CzIPN alone is not sufficient to enhance HER activity in this catalytic system. The lower activity may arise from changes in the local organization, accessibility, or photophysical behavior of the catalytic components, but the available data do not allow a definitive assignment.

**FIGURE 3:**
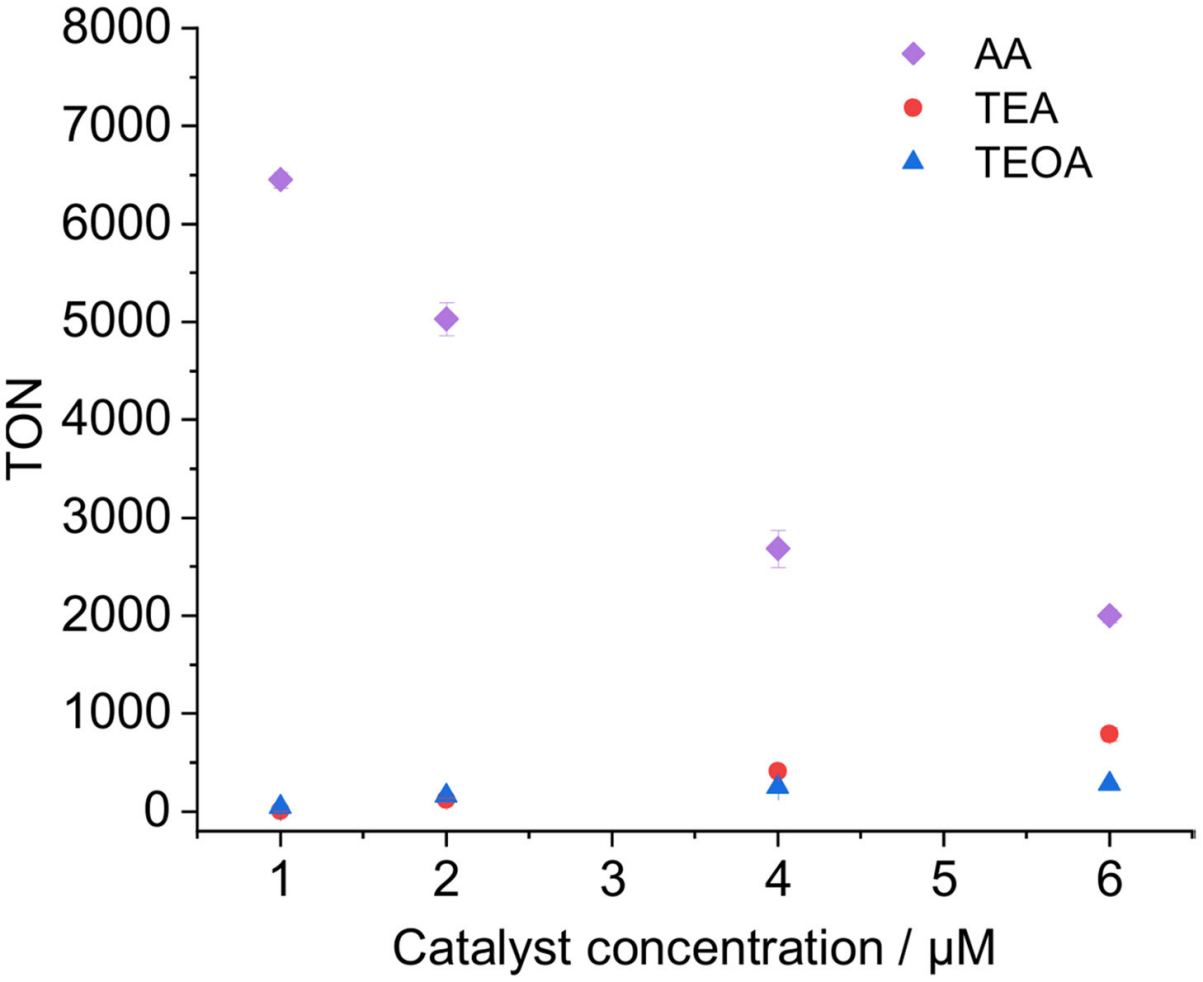
Light‐driven HER with ammonium ascorbate (AA), triethylamine (TEA), and triethanolamine (TEOA) as SEDs at various catalyst concentrations over 20 h of irradiation (*λ* = 453 nm, TON values calculated with respect to the catalyst, performed in triplicate, with error bars).

Further variations of the catalyst concentration and SED were performed under the optimized MeCN/H_2_O solvent conditions (Figure 3, Table S13). The complete screening data are summarized in Table S13. Lowering the cobaloxime concentration from 2 to 1 *µ*M increased the catalyst‐normalized TON from 5030 ± 166 to 6451 ± 82 after 20 h. In contrast, increasing the catalyst concentration to 4 or 6 µM led to lower TON values of 2688 ± 189 and 2001 ± 66, respectively. This trend indicates that a higher catalyst loading does not improve the catalyst‐normalized performance of the system under the applied conditions.

The identity of the SED had an even stronger influence on the HER activity. Replacing ammonium ascorbate with TEA or TEOA resulted in substantially lower TON values across the catalyst concentrations tested (Figure 3 and Table S13). The highest TON obtained with TEA was 790 ± 69 at 6 µM cobaloxime, whereas TEOA gave a maximum TON of 281 ± 7 at 6 µM cobaloxime. These values are far below those obtained with ammonium ascorbate. This comparison shows that ammonium ascorbate is particularly well suited for the present 4CzIPN/cobaloxime system. The difference may be related to the fact that ammonium ascorbate allows the reaction to be performed under mildly acidic conditions, whereas TEA and TEOA are typically used under more basic conditions, as reflected by the p*K*_a_ values of their conjugate acids (10.7 and 7.9, respectively) [17]. However, the activity differences should not be attributed to pH alone, since proton availability, catalyst speciation, donor oxidation kinetics, and the efficiency of 4CzIPN quenching may all contribute to the observed performance.

**FIGURE 4:**
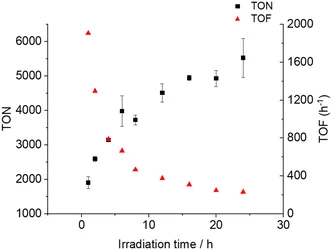
Time course of light‐driven HER under optimized conditions, showing the increase in TON and the corresponding decrease in TOF during irradiation. Reaction conditions: 0.1 mM 4CzIPN, 1 µM cobaloxime, 1 mg/mL dmgH_2_, and 0.1 M ammonium ascorbate in MeCN/H_2_O containing 20 vol.% H_2_O (irradiation at 453 nm; TON and TOF values were calculated with respect to the catalyst; experiments were performed in triplicate, with error bars).

A time‐course experiment was then performed under the best conditions identified in the screening, using 0.1 mM 4CzIPN, 1 µM cobaloxime, 1 mg/mL dmgH_2_, and 0.1 M ammonium ascorbate in MeCN/H_2_O containing 20 vol.% H_2_O (Figure 4). Hydrogen evolution was fastest during the initial phase of irradiation, with an average TOF of 1906 h^−1^ over the first hour. The average TOF decreased to 786 h^−1^ after 4 h and to 230 h^−1^ over 24 h, indicating progressive deactivation of the catalytic system. After 24 h, hydrogen evolution had largely plateaued.

No visible color change of the reaction mixture was observed after irradiation. UV/Vis spectra recorded from the reaction solution before and after light‐driven HER showed no pronounced decrease of the 4CzIPN absorption around 440 nm (Figure S4), suggesting that the PS remains largely intact under these conditions. However, these UV/Vis data do not directly prove the integrity of the cobalt catalyst. The observed loss of activity is therefore more consistent with deactivation of the cobaloxime catalyst than with degradation of 4CzIPN, although this assignment remains indirect. Such deactivation is plausible, as cobaloxime catalysts are known to undergo ligand dissociation and decomposition under photocatalytic conditions [9, 37, 42].

Control experiments confirmed that all components are required for efficient hydrogen evolution. No H_2_ production was detected when either the catalyst, the PS, or dmgH_2_ was omitted. In the absence of the SED, the combination of 4CzIPN and cobaloxime gave only a very low TON of 5.4 ± 0.59 after 20 h. This value is negligible compared with the optimized system and confirms that ammonium ascorbate is required for productive HER under the applied conditions.

Overall, the optimized 4CzIPN/cobaloxime system reached a TON of 6451 ± 82 after 20 h irradiation. This value demonstrates that 4CzIPN can act as an efficient organic PS for cobaloxime‐catalyzed light‐driven HER using ammonium ascorbate as the SED. Under the optimized conditions of 0.1 mM 4CzIPN, 1 µM cobaloxime, 1 mg/mL dmgH_2_, and 0.1 M ammonium ascorbate in MeCN/H_2_O containing 20 vol.% H_2_O, this corresponds to an apparent quantum yield (AQY) of *φ* = 0.123% (Sections S6 and S8). Comparison with previously reported PS‐cobaloxime systems remains challenging because the reported activities strongly depend on irradiation time, solvent composition, SED, catalyst loading, PS loading, and the basis used for TON calculation. Nevertheless, the present system compares favorably when TON is evaluated with respect to the catalyst concentration, while some previously reported systems remain superior when the performance is normalized to the PS concentration or when longer irradiation times are considered (Table S14) [9, 14, 42].

### 2.3 Mechanistic Insights

**FIGURE 5:**
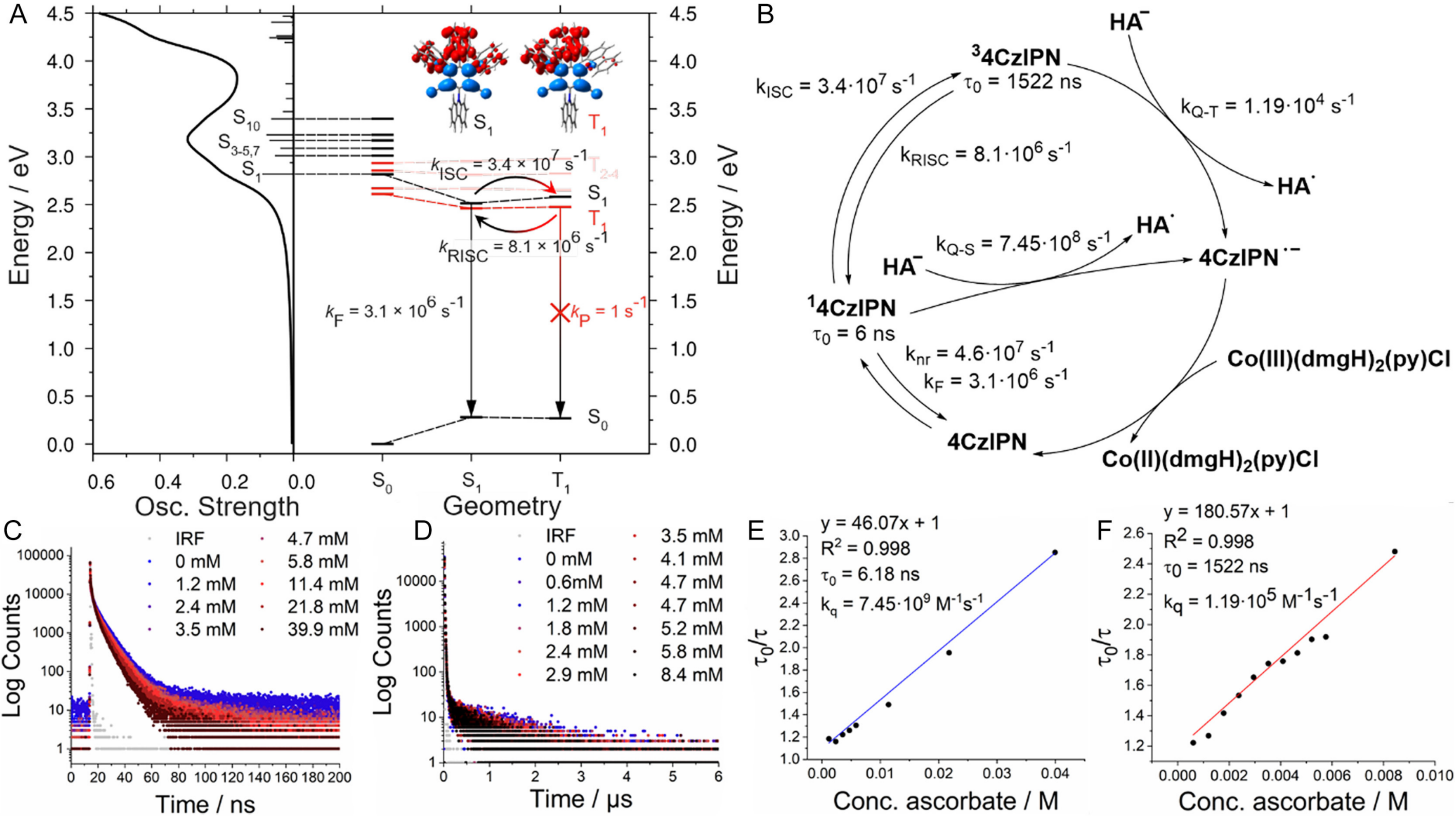
(A) Simulated excited‐state energies and processes involved in the initial photoexcitation of 4CzIPN (left) and kinetics of the subsequent intramolecular processes (right) associated with comparably fast intersystem crossing (ISC) from the thermally relaxed S_1_ state to the lowest‐energy triplet state (T_1_), as well as reverse ISC (RISC); see charge‐density difference plot as obtained within the respective fully relaxed S_1_ and T_1_ geometries (available via Zenodo). Radiative rates, i.e., fluorescence from relaxed S_1_ and phosphorescence from equilibrated T_1_ are given. (B) Reductive quenching mechanism of 4CzIPN within the photocatalytic cycle, displaying the competing processes with rate constants. Luminescence decays of the prompt fluorescence (C) and delayed fluorescence (D) of 4CzIPN with increasing amounts of quencher (ammonium ascorbate) and the corresponding Stern–Volmer plots (E,F).

To gain a deeper understanding of the interaction between the PS (4CzIPN), the catalyst (cobaloxime), and the SED (ammonium ascorbate), Stern–Volmer quenching studies were performed (Tables S15 and S16 for details). Since ammonium ascorbate was used in 50,000 equivalents in comparison to the catalyst, reductive quenching of the excited‐state of the PS 4CzIPN* by the catalyst can be neglected, and quenching by the ascorbate is likely to be the dominant reaction path. Due to the close first excited singlet‐triplet energy gap of 4CzIPN, there are two excited states (S_1_ and T_1;_ for simulated absorptions see Table S1), which could both be quenched (Figure 5A,B). After optical excitation to the S_1_ state, the T_1_ state is populated through ISC on a time scale of 10^−8^ s for 4CzIPN. The Stern–Volmer quenching constants for the singlet and triplet state are usually not the same, typically, *K*_SV_^T^ is higher than *K*_SV_^S^, because of the much longer lifetime of the former [43]. For this reason, TADF compounds usually do not yield a linear Stern–Volmer plot when *I*_0_/*I* is plotted against the concentration of the quencher [43].

At low concentrations of the quenching agent, the T_1_ state can be populated and is then efficiently quenched (Figure 5A). When the concentration of the quencher is increased, ISC to the T_1_ state is outcompeted by quenching of the S_1_ state; however, due to its lower Stern–Volmer constant, this happens less efficiently. This results in a Stern–Volmer plot that features a high slope in the beginning at low quencher concentration, and then flattens out at higher concentrations [43]. To better distinguish between the two processes, we measured fluorescence lifetime in the presence of a quencher by time‐correlated single‐photon counting (TCSPC) spectroscopy under strictly oxygen‐free conditions (Figure 5C,D).

The Stern–Volmer constant (*K*_SV_) for PF quenching was found to be 46 and that for DF quenching was 181 (Figure 5E,F), which we assume to be *K*_SV_^S^ and *K*_SV_^T^, respectively. From these, the bimolecular quenching constants (*k*_q_) were calculated using Formula (1) [44]

(1) $$k_{q}=\frac{K_{\mathrm{SV}}}{\tau_{0}}$$

where *k*_q_: biomolecular quenching constant/M^–1^ s^–1^; *K*_SV_: Stern‍–‍Volmer constant/M^–1^; *τ*_0_: lifetime without quencher/s.

We obtained *k*_q_ = 7.5 · 10^9^ M^−1^ s^−1^ for the bimolecular quenching constant of the singlet excited state, which is close to the diffusion‐controlled limit of the quenching rate of ca. 1 · 10^10^ M^−1^ s^−1^ [33]. The bimolecular quenching constant for the triplet state (1.2 · 10^5^ M^−1^ s^−1^) is significantly lower. This indicates that quenching of the singlet state happens more efficiently than quenching of the triplet state, despite the higher *K*_SV_^T^.

With the given concentration of the quencher ammonium ascorbate (*c*_q_ = 0.1 M), a rate constant can be calculated using Formula (2) [45].

(2) $$k_{Q}=k_{q}\cdot c_{q}$$

where *k*_Q_: rate constant for quenching/s^–1^; *k*_q_: biomolecular quenching constant/M^–1^ s^–1^; *c*_q_: concentration of the quencher/M.

The calculated rates are displayed together with the rates for ISC, RISC, and nonradiative decay from the singlet state (*k*_nr_) in acetonitrile in Figure 5B [46].

In order to provide a detailed understanding of the electronic processes related to the population transfer between the low‐lying singlet and triplet states as well as their kinetics, we performed additional SR TDDFT simulations. To this aim, the S_1_ and T_1_ states were fully relaxed at the (TD)DFT level(s) of theory. Subsequently, the energetics of the low‐lying singlet and triplet states as well as their spin–orbit couplings (SOCs) were analyzed within the Franck–Condon point (S_0_ geometry) as well as in the S_1_ and T_1_ structures (Figure 5A and Tables S2–S4). Within all three equilibrium geometries, T_1_ lies by 0.21, 0.05, and 0.10 eV energetically lower than the S_1_ state, while the SOC is, with 0.4 cm^−1^, rather small—as required in case of TADF emitters [22, 47, 48]. Notably, the$\left\langle T_{1}|{\hat{H}}_{\mathrm{SOC}}|S_{1}\right\rangle$—the SOC between S_1_ and T_1_—decreases to 0.1 cm^−1^ within the S_1_ and 0.3 cm^−1^ within the T_1_ structures, respectively. Based on the respective energetics as well as the SOCs, rate constants for ISC and RISC were calculated (Table S5). Upon considering structural reorganization, ISC (energetically downhill) occurs with a rate of 3.4 · 10^7^ s^−1^, and RISC (energetically uphill) occurs more slowly with 8.1 · 10^6^ s^−1^. With 3.1 · 10^6^ s^−1^, fluorescence from S_1_ is slightly slower than RISC, which allows to build up an S_1_ population, followed by ISC and subsequent RISC. In contrast, phosphorescence is—due to the almost negligible transition‐dipole moment between the lowest energy spin–orbit states (a linear combination of the respective spin‐free singlet and triplet states, see Supporting Information for details)—with 1 s^−1^ several orders of magnitude slower and thus irrelevant for 4CzIPN.

In addition to these (first order) intramolecular kinetics, branching ratios for the triplet and singlet quenching were calculated using Formulas (3) and (4) to estimate which quenching pathway is more important. In doing so, the rate of interest is divided by the sum of all rates of the possible competing processes (see Figure 5B). The rate constant of the nonradiative decay of 4CzIPN from S_1_ to S_0_ in acetonitrile was obtained from the literature (*k*_nr_ = 4.6 · 10^7^ s^−1^) [46].

(3) $$\Phi_{\mathrm{Q-S}}=\frac{k_{\mathrm{Q-S}}}{k_{\mathrm{Q-S}}+k_{\mathrm{ISC}}+k_{\mathrm{nr}}+k_{F}}$$

where *ϕ*_Q–S_: branching ration for singlet quenching; *k*_Q–S_: rate for singlet quenching/s^–1^; *k*_ISC_: rate for intersystem crossing/s^−1^; *k*_nr_: rate for nonradiative decay/s^−1^; *k*_F_: rate for fluorescence/s^−1^.

(4) $$\Phi_{\mathrm{Q-T}}=\frac{k_{Q-T}}{k_{\mathrm{Q-T}}+k_{\text{RISC}}}$$

where ϕ_Q–T_: branching ration for triplet quenching; *k*_Q–T_: rate for triplet quenching/s^–1^; *k*_RISC_: rate for reverse intersystem crossing/s^−1^.

Values of *Φ*_Q‐S_ = 90% and *Φ*_Q‐T_ = 0.2% demonstrate that quenching of the singlet state is by far the dominant pathway, while quenching of the triplet state can be neglected. Our findings therefore contradict the assumption that TADF compounds perform excellently as PSs due to their long‐lived triplet state [49]. Yu and coworkers found a similar effect for 4CzIPN with TEA as SED in the photocatalytic water reduction. In this system, the reductive quenching step took 0.2–0.3 ns and was faster than ISC [14]. The different quenching efficiencies between singlet and triplet state in TADF compounds were recently rationalized by the difference between the respective hole‐electron density distributions and a lower cage‐escape yield for the S_1_ state [50, 51].

In addition, the anthraquinone‐based PS DAHA reported by Ming et al. features only a very short excited‐state lifetime of 0.59 ns. However, due to the highly efficient quenching with 1,3‐dimethyl‐2‐phenyl‐2,3‐dihydro‐1*H*‐benzo[*d*]imidazole (BIH) (*k*_q_ = 1.2 · 10^10^ M^−1^ s^−1^) enormous TONs of up to 780,000 in HER were obtained after 160 h [9]. From this, it can be concluded that for photosensitizers, one should not only focus on the long lifetime of an excited state but rather more on the interaction between the SED as quencher and the PS to achieve efficient quenching and light‐driven HER.

## 3 Conclusions

In conclusion, we could show that 4CzIPN can act as an efficient organic PS for the cobaloxime catalyst in the light‐driven hydrogen evolution. In contrast to previous studies using 4CzIPN for photocatalytic hydrogen evolution with a palladium catalyst, the present system combines the noble‐metal‐free TADF PS 4CzIPN with the molecular cobalt catalyst cobaloxime and ammonium ascorbate as SED. Systematic optimization of the solvent composition, PS concentration, catalyst concentration, and SED identified MeCN/H_2_O containing 20 vol.% H_2_O, 0.1 mM 4CzIPN, 1 µM cobaloxime, 1 mg/mL dmgH_2_, and 0.1 M ammonium ascorbate as the best conditions. Under these conditions, a TON of 6451 ± 82 was obtained after 20 h irradiation. To the best of our knowledge, this represents the highest catalyst‐normalized TON reported so far for hydrogen evolution using a TADF PS.

The additional optimization experiments also showed that ammonium ascorbate is substantially more effective than TEA or TEOA in the present 4CzIPN/cobaloxime system. Furthermore, the addition of PDha‐g‐PEG did not improve HER performance under the conditions tested, indicating that improved dispersion of 4CzIPN alone is not sufficient to enhance activity in this system. Time‐course experiments revealed that hydrogen evolution is fastest during the initial phase of irradiation and progressively slows down, with the average TOF decreasing from 1906 h^−1^ after 1 h to 230 h^−1^ after 24 h. UV/Vis measurements before and after light‐driven HER suggest that 4CzIPN remains largely intact, whereas the loss of activity is more likely related to deactivation of the cobaloxime catalyst.

In addition to the catalytic optimization, we investigated the photocatalytic mechanism with Stern–Volmer quenching experiments and SR quantum chemical calculations. With TCSPC spectroscopy, we were able to distinguish between prompt and DF quenching, which corresponded to quenching of the S_1_ and T_1_ states, respectively. We found that the main quenching pathway of 4CzIPN* is via the S_1_ state and not via the T_1_ state. This is mainly because the rate constant of singlet quenching (*k*_Q‐S_ = 7.45 · 10^8^ s^−1^) is one order of magnitude higher than the rate constant of ISC, and the rate constant of RISC (*k*_RISC_ = 8.1 · 10^6^ s^−1^) is even two orders of magnitude higher than the rate constant of triplet quenching. This in‐depth investigation of the quenching mechanism allows for a better understanding of the interaction between each component and will help to optimize this catalytic system for future applications by choosing the right SED for each PS to maximize quenching efficiency.

## Funding

This work was supported by the Deutsche Forschungsgemeinschaft (project number 364549901).

## Conflicts of Interest

The authors declare no conflicts of interest.

## Supporting information

Supplementary Material

## Data Availability

The data obtained in this study have been deposited in the repository Zenodo and are available under the digital object identifier: https://doi.org/10.5281/zenodo.17734054
