# Supplementary material for: 4CzIPN Photosensitizes the Cobaloxime‐Catalyzed Light‐Driven Hydrogen Evolution Reaction
Source: ChemSusChem. 2026 Aug 22;19(16):e70942. doi: 10.1002/cssc.70942 (PMC13499262; doi:10.1002/cssc.70942)
Supplement: Supplementary file 1 [file CSSC-19-e70942-s001.pdf]

4CzIPN Photosensitizes the Cobaloxime-catalyzed Light-Driven Hydrogen Evolution Reaction

Alexander Tombrink,+[a] Benedikt Callies,+[b] Stephan Kupfer,[c] Daniel Kowalcyk,[d] Benedikt Wiedemann,[d] Dirk Ziegenbalg,[d] Kalina Peneva*[b, e, f] and Birgit Esser*[a,g]

[a] Institute of Organic Chemistry II and Advanced Materials, Ulm University, Albert-Einstein-Allee 11, 89081 Ulm, Germany. Email: Birgit.esser@uni-ulm.de

[b] Institute of Organic Chemistry and Macromolecular Chemistry, Friedrich Schiller University Jena, Lessingstraße 8, 07743 Jena, Germany. Email: kalina.peneva@uni-jena.de

[c] Institute of Physical Chemistry, Friedrich Schiller University Jena, Helmholtzweg 4, 07743 Jena, Germany

[c] Institute of Chemical Engineering, Ulm University, Albert-Einstein-Allee 11, 89081 Ulm, Germany

[e] Center for Energy and Environmental Chemistry Jena (CEEC Jena), Philosophenweg 7, Jena, 07743, Germany

[f] Jena Center for Soft Matter (JCSM), Friedrich Schiller University Jena, Philosophenweg 7a, Jena,

07743, Germany

[g] CELEST Green Energy Lab Ulm, Ulm University, Lise-Meitner-Str. 16, 89081 Ulm, Germany

+ These authors contributed equally

Table of Contents

1 Materials and Methods S3
1.1 Chemicals and Solvents S3
1.2 UV/Vis Absorption Spectra S3
1.3 Fluorescence Spectra S3
1.4 Time-correlated Single-Photon Counting S3
1.5 Cyclic Voltammetry S3
1.6 UV/Vis Spectroelectrochemistry (SEC) S4
1.7 Excited State Redox Potential S4
2 Time-correlated Single-Photon Counting S5
3 Cyclic Voltammograms S5
4 UV/Vis Spectroelectrochemistry S6
5 Computational Details and Results S6
5.1 4CzIPN Photosensitizer S8
5.2 Cobaloxime Catalyst S15
6 Radiometry S18
7 Light-driven hydrogen evolution S20
8 AQY Calculation S28
9 Stern-Volmer Quenching Experiments S29
10 References S31

1 Materials and Methods

1.1 Chemicals and Solvents

Chemicals were purchased from Roth, Sigma-Aldrich, BLD Pharm or VWR and used directly without further purification unless otherwise noted. Anhydrous solvents (CH_3CN, CH_3OH) were obtained from VWR and Sigma-Aldrich. The photosensitizer 4CzIPN was purchased from BLD Pharm and the cobaloxime catalyst was obtained from Sigma-Aldrich.

1.2 UV/Vis Absorption Spectra

UV/Vis absorption spectra were measured on a SHIMADZU UV-3600i Plus UV-VIS-NIR spectrophotometer using Quartz cuvettes (1 cm path length) from HELLMA ANALYTICS.

1.3 Fluorescence Spectra

Fluorescence spectra were measured on a PERKINELMER LS 55 luminescence spectrometer using Quartz cuvettes (1 cm path length) from HELLMA ANALYTICS.

1.4 Time-correlated Single-Photon Counting

Fluorescence lifetimes were recorded with a DeltaPro from Horiba Scientific using a 481, 451 or 372 nm pulsed Laser source (Class 3B Laser Product, <0.5 W peak in pulsed and CW mode). The Delta Pro consists of: DeltaDiode (Picosecond diode controller), DeltaHub (High throughput TCSPC controller), DPS-1 (Detector Power supply) and a PPD (Picosecond photon detection module). The IRF (Instrument response function) was measured with LUDOX silica nanoparticles. Fits were done with the software DecayFit – Emission Decay Analysis Software (Version 1.4).

1.5 Cyclic Voltammetry

Cyclic voltammograms were measured in anhydrous degassed MeCN solution in an argon-filled glovebox using a METROHM Autolab PGSTAT128N. As the working electrode, a glassy carbon disc electrode (2 mm in diameter) was used, and a platinum rod served as the counter electrode. As the reference electrode, an $Ag/AgNO_3$ electrode containing a silver wire immersed in an inner chamber filled with 0.1 M $AgNO_3$ and an outer chamber containing 0.1 M n-Bu_4NPF_6 were used. For internal reference, the ferrocene/ferrocenium (Fc/Fc^+) redox couple was used.

1.6 UV/Vis Spectroelectrochemistry (SEC)

UV/Vis Spectroelectrochemistry (SEC) was measured in an optically transparent thin-layer electrochemical cell (OTTLE cell) with PtPtAg/POM module and CaF_2 windows in acetonitrile (Pt mesh working electrode, Pt mesh counter electrode, Ag wire pseudo reference electrode).[1] UV/Vis absorption spectra were measured on a SHIMADZU UV-3600i Plus UV-VIS-NIR spectrophotometer.

1.7 Excited State Redox Potential

The excited state redox potential was calculated by the following formula:[2]

$E^*_{1/2\text{-red}}(\mathrm{PS^*/PS^{\bullet-}}) = E_{1/2\text{-red}}(\mathrm{PS^*/PS^{\bullet-}}) + E_{0,0}$

The zero-zero vibrational state excitation energy $E_{0,0}$ was estimated by the energy at which the absorption and emission spectra overlap.

2 Time-correlated Single-Photon Counting

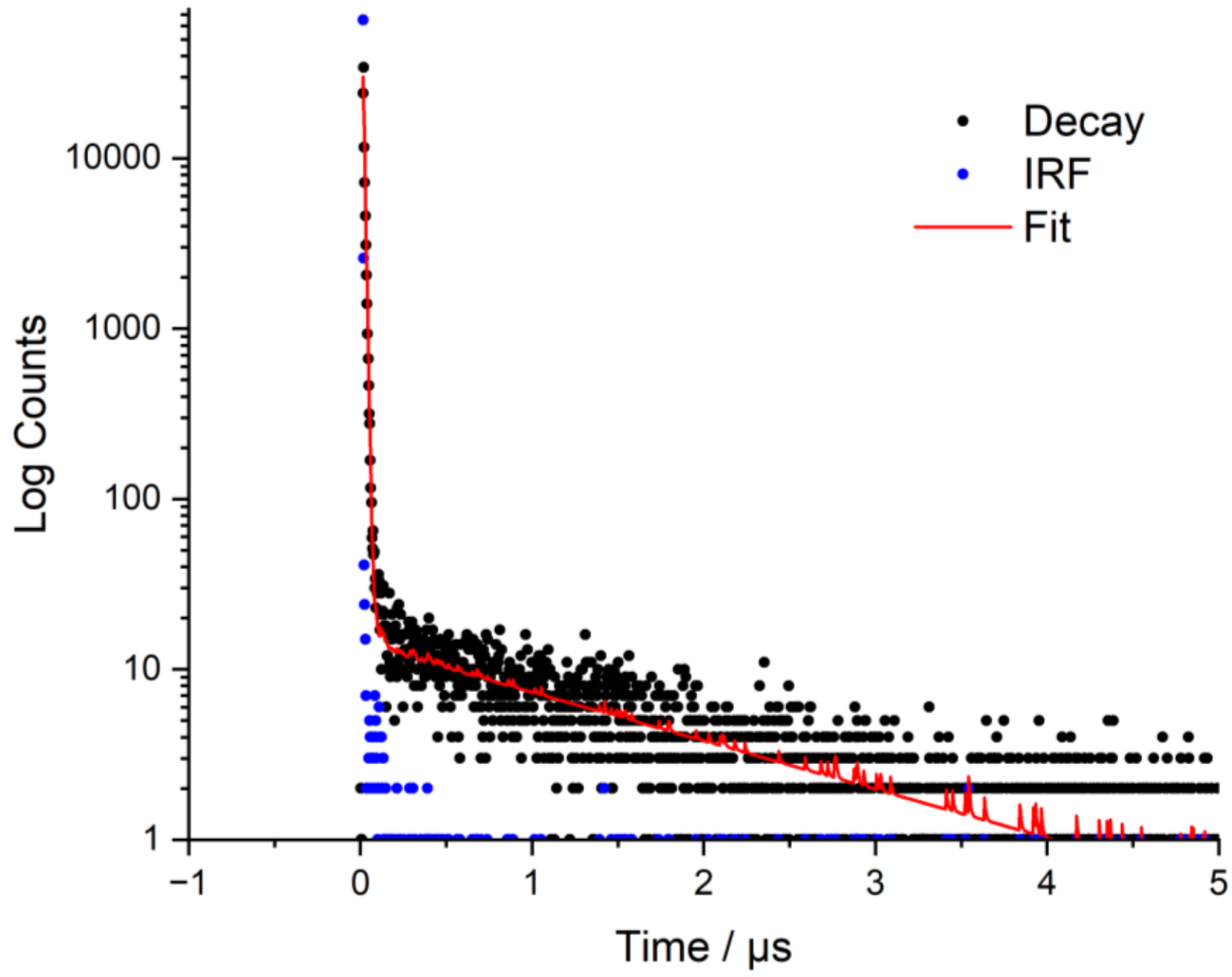

Figure S1: Emission decay spectrum of 4CzIPN in a MeCN / H_2O (1/1, v/v) solution (TCSPC, degassed with argon).

3 Cyclic Voltammograms

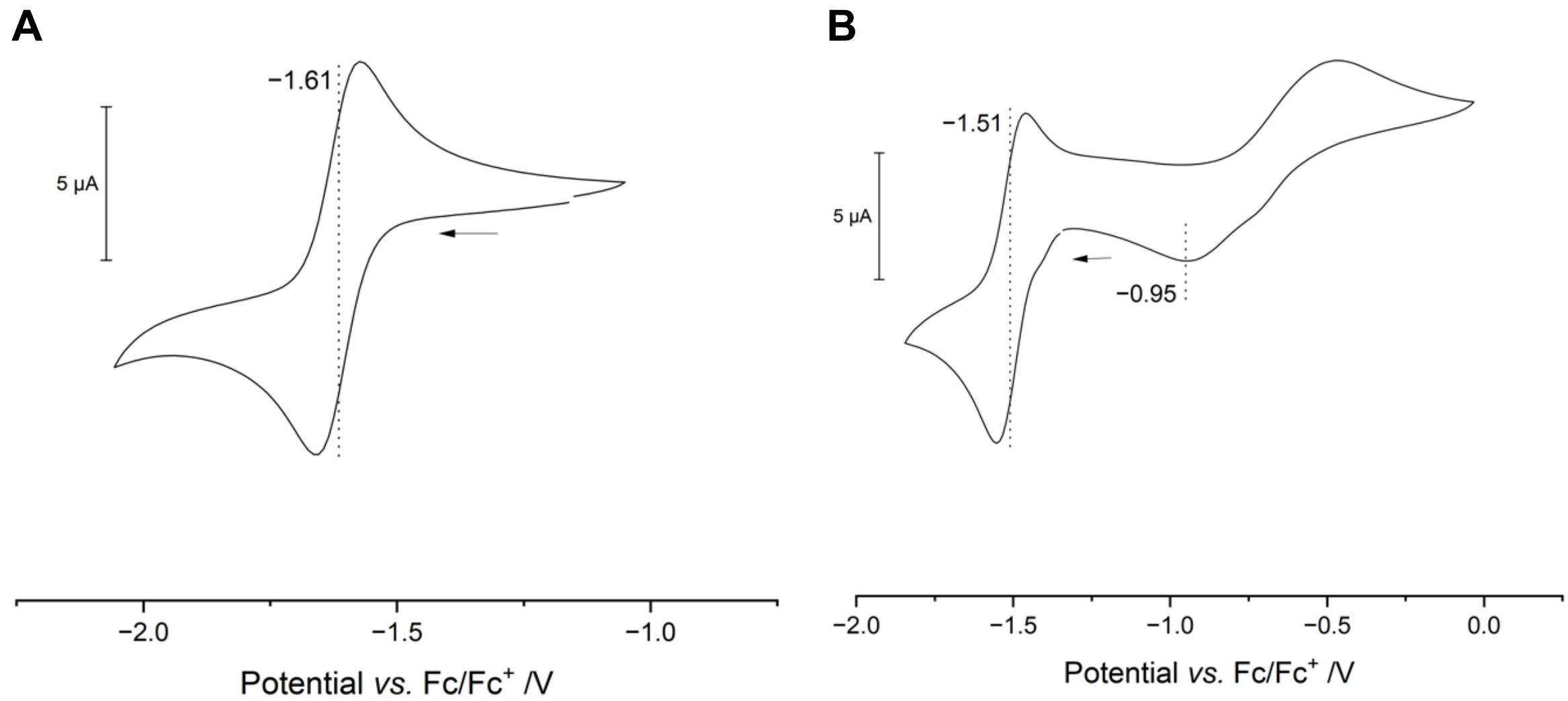

Figure S2: Cyclic voltammograms of 4CzIPN (A) and cobaloxime (B) recorded in MeCN containing 0.1 *M* n-Bu_4NPF_6 as supporting electrolyte. Analyte concentration: 1 m*M*; working electrode: glassy carbon; counter electrode: platinum rod; reference electrode: $Ag/AgNO_3$. Potentials are reported versus Fc/Fc^+.

4 UV/Vis Spectroelectrochemistry

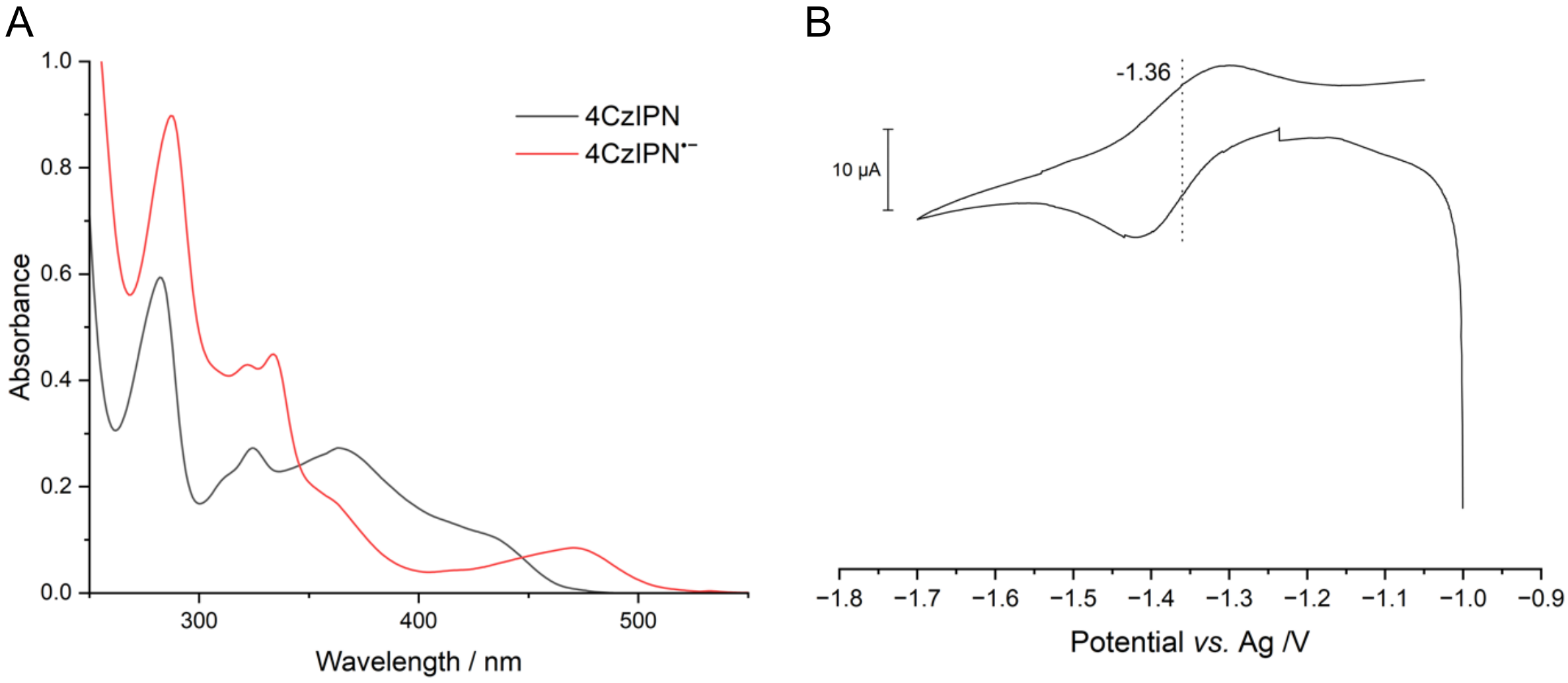

Figure S3: UV/Vis spectroelectrochemistry of 4CzIPN in MeCN. A) UV/Vis absorption spectra of neutral 4CzIPN and electrochemically generated $4CzIPN^{\bullet-}$. The radical anion was generated by applying a potential of −1.45 V in the OTTLE cell. B) Cyclic voltammogram of 4CzIPN recorded in the OTTLE cell in MeCN containing 0.2 M *n*-Bu_4NPF_6. Analyte concentration: 1 mM.

5 Computational Details and Results

All quantum chemical simulations to address structural properties of **4CzIPN** (photosensitizer, PS) as well as of the cobaloxime catalyst (CAT) were performed using the Gaussian 16 software package.[3] To this aim density functional theory, i.e. the PBE0[4] hybrid function in combination with the def2-svp basis set[5], was utilized. Implicit solvent effects (acetonitrile) were taken into account by SMD[6,7]; in case of geometry optimizations the equilibrium solvent model was used. Furthermore, D3 dispersive correction was applied in combination with Becke-Jones dumping (D3BJ).[8] In case of the PS, the singlet ground state (S_0) as well as the triplet ground state (T_1) equilibrium structures were optimized using DFT. Furthermore, the S_1 equilibrium structure was relaxed using time-dependent (TD)DFT with an analog setup as in case of the previous ground state simulations. For the CAT, the non-reduced singlet species, i.e. Co(III), was optimized using DFT, while the singly reduced cobalt complex was investigated in doublet (D_0) and quartet multiplicity (Q_0), while formation of the respective doublet species is thermodynamically favored (see Table S7). Upon the second reduction event, dissociation of the chloride ligand occurs within the thermodynamically preferred singlet species, due to the doubly occupied $\sigma_{z^2}^*$ orbital. This pyramidal Co(I) complex features a d^8 configuration; see Table S7 for details.

Frequency calculations were performed to verify the presence of (local) minima on the 3*N*-6-dimensional hypersurface.

Subsequently, scalar-relativistic (SR-)TDDFT simulations were performed in Orca 6.0.1[9] and using the scalar-relativistic zeroth-order regular approximation (SR-ZORA). DFT and TDDFT calculations were performed using the PBE0 XC functional. All other atoms were described using the respective def2-TZVP basis sets (with the corresponding SARC/J auxiliary basis set).[10] The 50 lowest singlet-singlet and singlet-triplet excitations were obtained, while SOCs were obtained at the SR-ZORA-TDDFT level of theory. The effects of interaction with acetonitrile were taken into consideration at the CPCM (conductor-like polarizable continuum model) level of theory. (SR-)TDDFT simulations (including SOCs) were carried out within the previously optimized S_0, S_1 and T_1 equilibrium geometries. For the cobaloxime catalyst, such calculations were exclusively performed within the non-reduced singlet ground state (S_0).

Finally, the kinetics of intersystem crossing (ISC), reverse ISC (RISC) and the radiative component of the emissive processes (fluorescence and phosphorescence, respectively) were simulated based on the SR-TDDFT simulations. In case of energetically downhill ISC ($S_1 \rightarrow T_1$), the simplified rate equation with Fourier transformed Lorentzian dephasing was utilized[11]:

$$k_{ISC} = \frac{\gamma}{\hbar(E_{kl}^2 + \gamma^2)} |SOC|^2,$$

where γ represents half-width at half-maximum (0.2 eV used in all cases), $\hbar$ is the reduced Plank constant, E_{kl} and SOC are adiabatic energy difference and the spin-orbit coupling between two states ($SOC = \langle T_l | \hat{H}_{SOC} | S_k \rangle$). In the similar fashion, the RISC ($T_1 \rightarrow S_1$) rates were calculated using the following formula[11]:

$$k_{RISC} = \frac{\gamma}{\hbar(E_{kl}^2 + \gamma^2)} |SOC|^2 e^{-\frac{E_{kl}}{k_B T}},$$

where k_B is a Boltzmann constant and T is temperature (298.15 K for all calculations). ISC and RISC rates were calculated in a vertical fashion, using the singlet-triplet energy spitting within the respective S_0, S_1 or T_1 geometry as well as considering structural reorganization from the S_1 to the T_1 equilibrium geometries.

The rates of fluorescence (k_F) and phosphorescence (k_P) were calculated according to the Einstein coefficient A for spontaneous emission[12,13]:

$$A = k_{F,P} = \frac{\omega^3}{3\epsilon_0 \pi \hbar^4 c^3} \mu^2,$$

where, ω denotes the resonance frequency of $S_1 \rightarrow S_0$ or $T_1 \rightarrow S_0$ the transition respectively, c is the speed of light in vacuum and ϵ_0 is vacuum permittivity. μ is the respective transition dipole moment. In case of phosphorescence, the transitions dipole moment for the (formally) spin-forbidden singlet and triplet transition was obtained by means of the respective spin-orbit states, which are a linear combination of the respective spin-free singlet and triplet states. Phosphorescence rates were calculated by summation over contributions from all three microstates of T_1 (m_s = -1, 0, 1).

Structural properties (XYZ files) as well as high resolution images of spin densities and CDDs plots are available free of charge via the Zenodo online repository (https://doi.org/10.5281/zenodo.17734054).

5.1 4CzIPN Photosensitizer

Table S1: Calculated electronic absorption bands of PS 4CzIPN as obtained at the B3LYP10/def2-svp level of theory in acetonitrile. Electronic properties of dipole-allowed singlet-singlet (left) and the 20 lowest-energy singlet-triplet transitions (right) are summarized, i.e., electronic characters, excitation energies, excitation wavelengths and oscillator strengths. Electronic characters are indicated by charge density difference plots, while charge transfer occurs from red to blue.

**Singlet-singlet**					**Singlet-triplet**			
Transition $S_0 \rightarrow S_i$	Character	ΔE_{0i} / eV	λ / nm	f_{0i}	Transition $S_0 \rightarrow T_i$	Character	ΔE_{0i} / eV	λ / nm
S_1	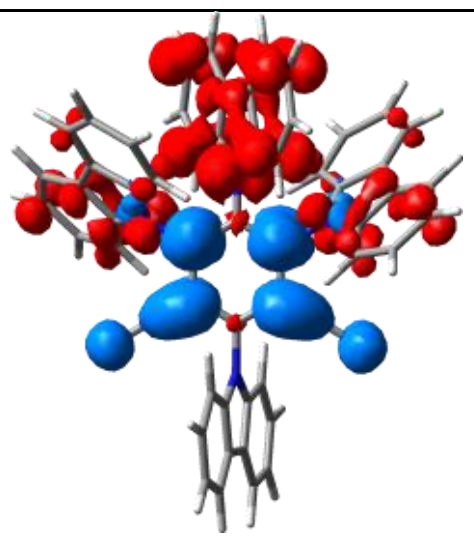	2.82	440	0.0935	T_1	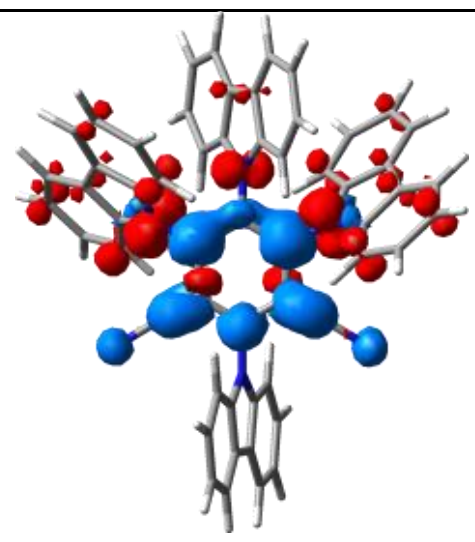	2.61	475

S_2	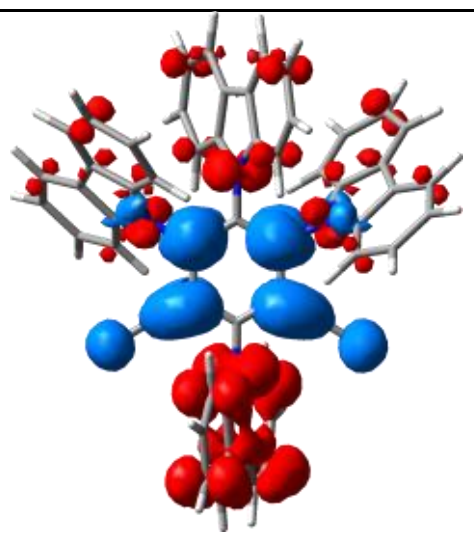	2.94	422	0.0115	T_2	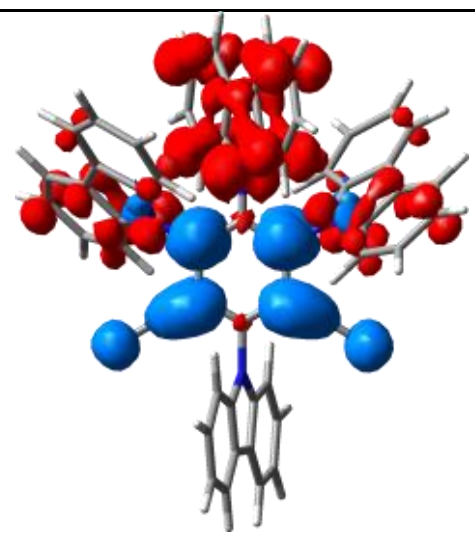	2.67	464
S_3	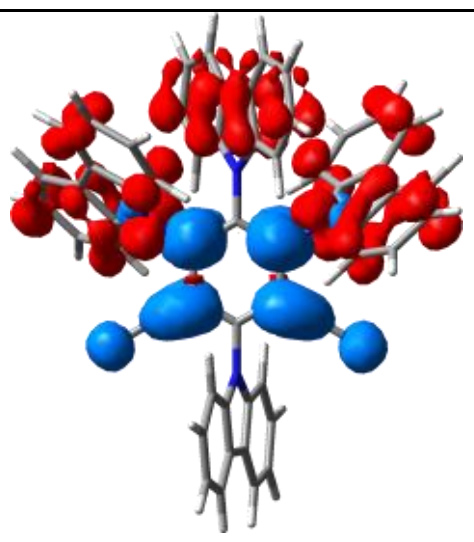	3.01	412	0.0560	T_3	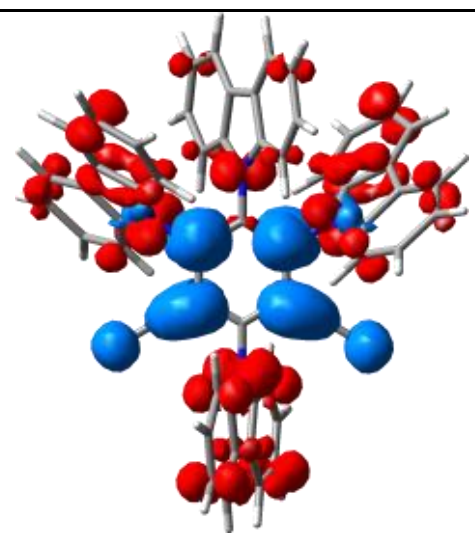	2.86	434
S_4	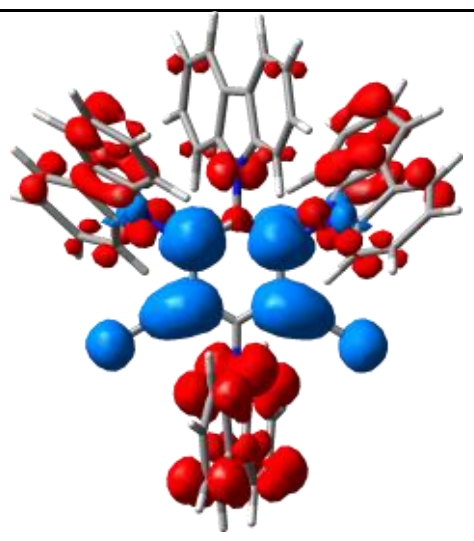	3.09	402	0.0397	T_4	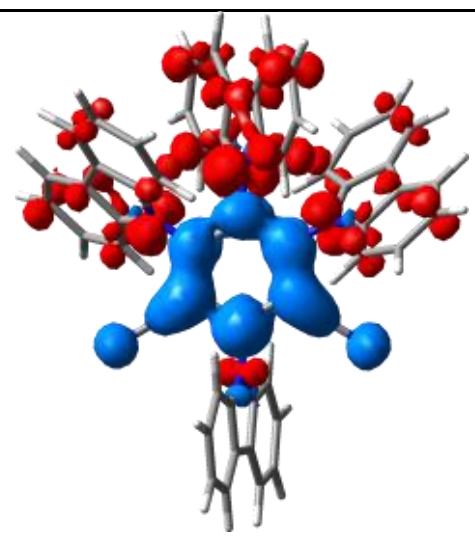	2.93	423
S_5	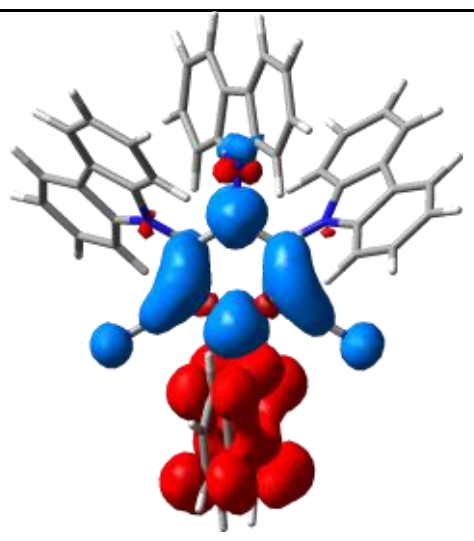	3.17	391	0.0721	T_5	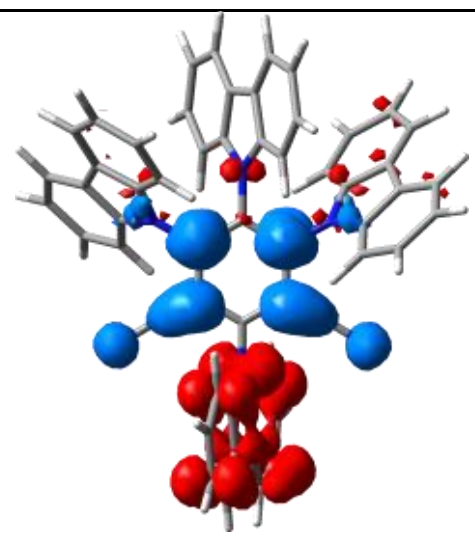	3.00	414
S_6	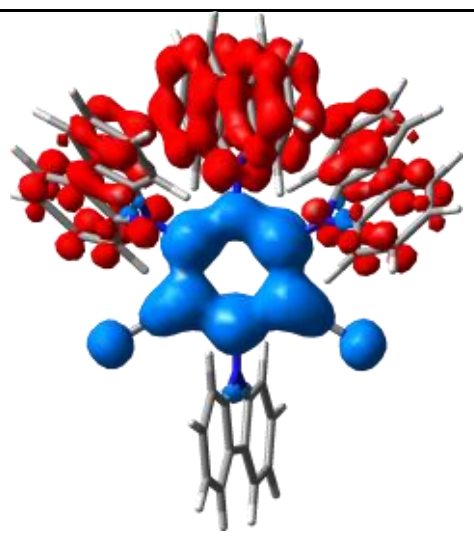	3.21	387	0.0034	T_6	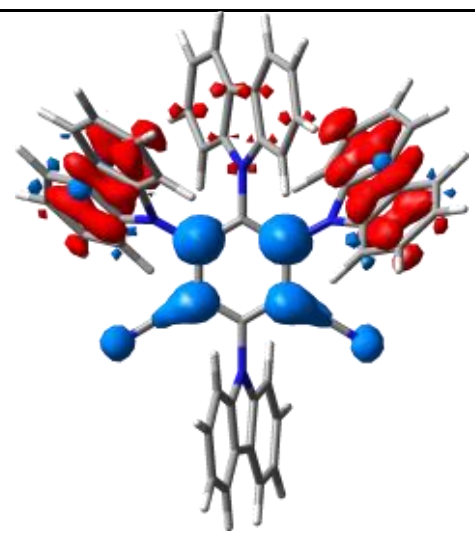	3.02	411
S_7	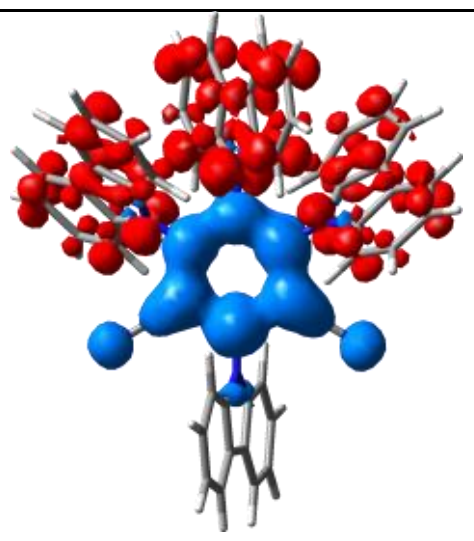	3.23	384	0.0811	T_7	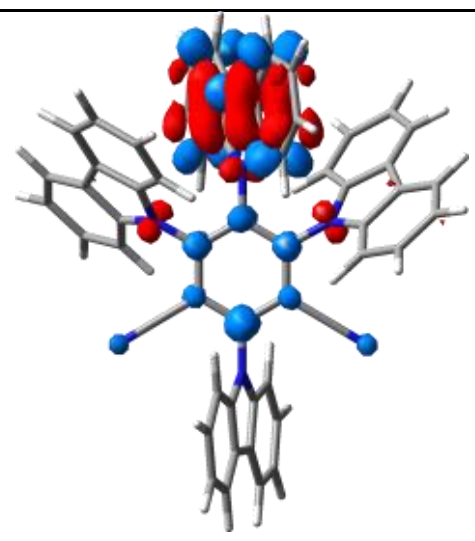	3.05	407

S_8	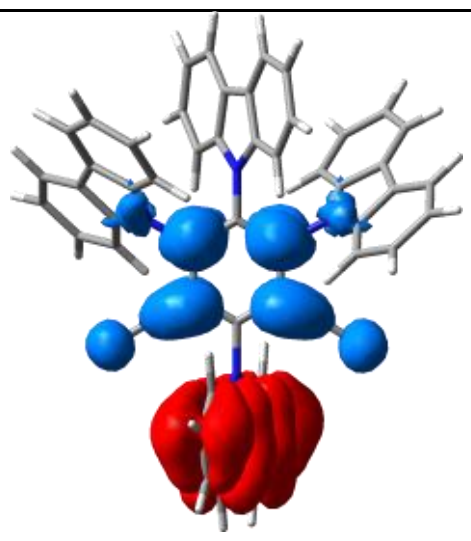	3.33	372	0.0015	T_8	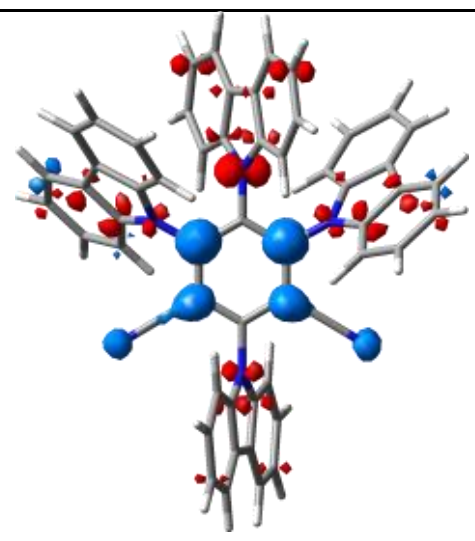	3.08	403
S_9	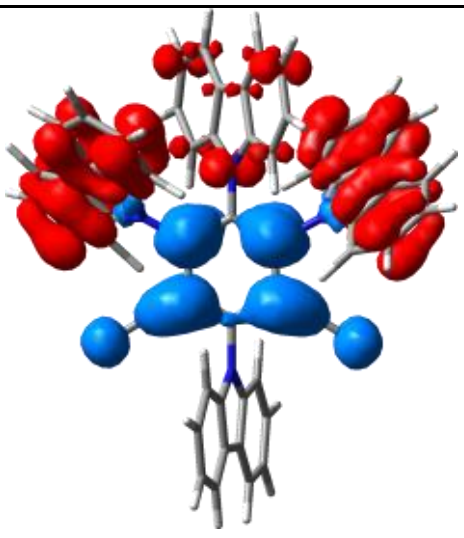	3.35	371	0.0018	T_9	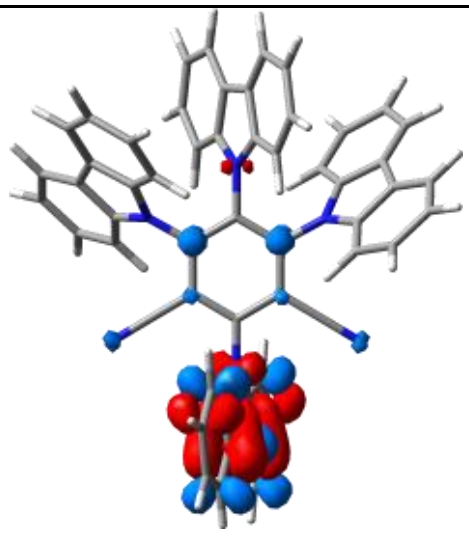	3.09	401
S_{10}	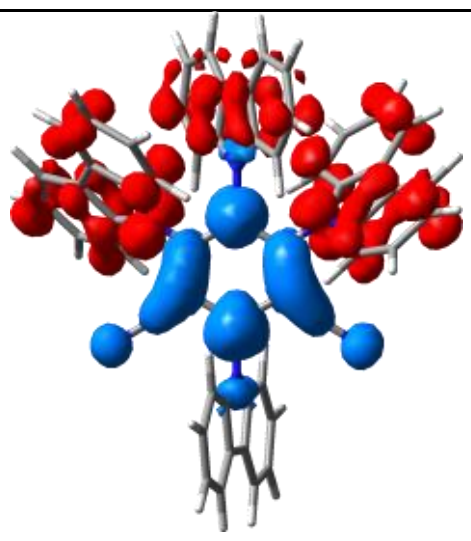	3.40	365	0.0666	T_{10}	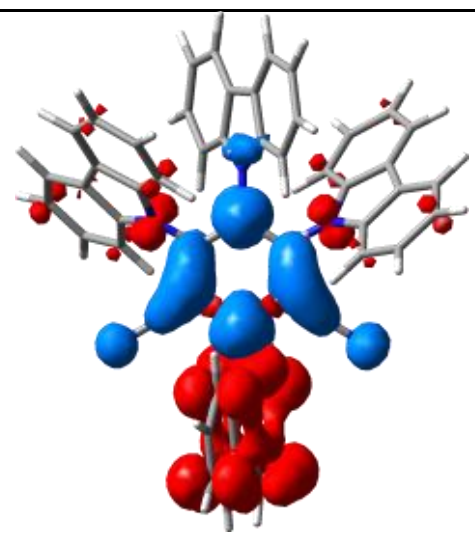	3.12	397
S_{35}	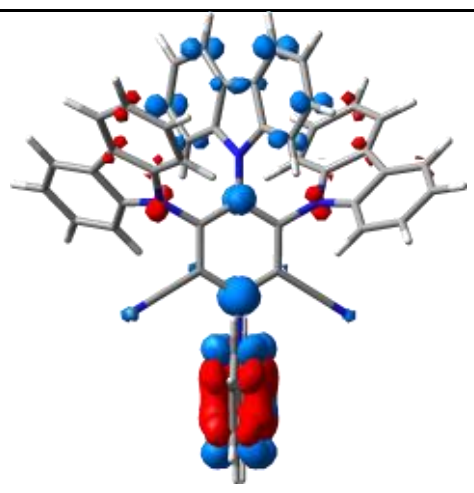	4.66	266	0.1780				

Table S2: Spin-orbit coupling matrix elements (SOCs in cm^{-1}) at the Franck-Condon point (S_0 geometry) between the eleven lowest singlet states (S_0 and S_1–S_{10}) and the ten lowest energy spin forbidden triplet states of PS 4CzIPN. Energies (in eV) and oscillator strengths of the respective singlet spin-free transitions (from S_0) are indicated. All energies are given with respect to the fully optimized singlet ground state energy.

			T_1	T_2	T_3	T_4	T_5	T_6	T_7	T_8	T_9	T_{10}
	f_{0i}	ΔE_{0i} / eV	2.61	2.67	2.86	2.93	3.00	3.02	3.05	3.08	3.09	3.12
S_0	-	0.00	0.1	0.7	3.9	1.0	0.7	0.1	0.2	0.7	0.1	3.0
S_1	0.0935	2.82	0.4	0.2	0.3	0.2	0.1	0.2	0.0	0.0	0.1	0.2
S_2	0.0115	2.94	0.4	0.3	0.1	0.4	0.3	0.2	0.1	0.2	0.1	0.2

S_3	0.0560	3.01	0.6	0.5	0.4	0.1	1.0	0.1	0.3	0.2	0.3	0.0
S_4	0.0397	3.09	0.8	0.3	0.6	0.7	0.2	0.2	0.1	0.2	0.0	0.2
S_5	0.0721	3.17	0.5	0.1	0.3	0.5	0.2	0.0	0.1	0.2	0.3	0.1
S_6	0.0034	3.21	0.3	0.2	0.1	0.4	0.3	0.0	0.1	0.2	0.2	0.2
S_7	0.0811	3.23	0.2	0.3	0.2	0.4	0.4	0.1	0.1	0.2	0.2	0.2
S_8	0.0015	3.33	0.1	0.2	0.1	0.0	0.1	0.0	0.0	0.2	0.3	0.0
S_9	0.0018	3.35	0.3	0.1	0.1	0.2	0.3	0.3	0.0	0.0	0.1	0.2
S_{10}	0.0666	3.40	0.6	0.1	0.0	0.5	0.1	0.3	0.2	0.0	0.0	0.7

Table S3: Spin-orbit coupling matrix elements (SOCs in cm^{-1}) at the S_1 geometry between the eleven lowest singlet states (S_0 and S_1–S_{10}) and the ten lowest energy spin forbidden triplet states of PS 4CzIPN. Energies (in eV) and oscillator strengths of the respective singlet spin-free transitions (from S_0) are indicated. All energies are given with respect to the fully optimized singlet ground state energy.

			T_1	T_2	T_3	T_4	T_5	T_6	T_7	T_8	T_9	T_{10}
	f_{0i}	ΔE_{0i} / eV	2.46	2.67	2.81	2.95	3.04	3.14	3.19	3.23	3.25	3.28
S_0	-	0.28	0.8	0.3	4.1	1.1	0.9	0.2	1.0	1.0	0.2	1.3
S_1	0.0257	2.51	0.1	0.6	0.4	0.1	0.4	0.1	0.0	0.1	0.1	0.2
S_2	0.0329	2.86	0.6	0.6	0.5	1.2	0.6	0.0	0.1	0.3	0.1	0.3
S_3	0.0067	2.87	0.4	0.4	0.2	0.6	0.4	0.1	0.2	0.2	0.1	0.2
S_4	0.0274	3.01	0.1	1.0	0.5	0.1	0.7	0.1	0.2	0.3	0.1	0.2
S_5	0.0001	3.17	0.1	0.1	0.1	0.1	0.2	0.0	0.2	0.1	0.0	0.1
S_6	0.0012	3.23	0.1	0.4	0.2	0.2	0.2	0.1	0.1	0.1	0.1	0.1
S_7	0.0105	3.24	0.1	0.3	0.1	0.1	0.5	0.0	0.1	0.1	0.1	0.2
S_8	0.0014	3.25	0.1	0.1	0.1	0.1	0.1	0.0	0.0	0.1	0.0	0.0
S_9	0.0085	3.29	0.1	0.1	0.2	0.3	0.0	0.1	0.1	0.1	0.0	0.0
S_{10}	0.0797	3.29	0.0	0.3	0.1	0.0	0.4	0.1	0.0	0.1	0.0	0.1

Table S4: Spin-orbit coupling matrix elements (SOCs in cm^{-1}) at the T_1 geometry between the eleven lowest singlet states (S_0 and S_1–S_{10}) and the ten lowest energy spin forbidden triplet states of PS 4CzIPN. Energies (in eV) and oscillator strengths of the respective singlet spin-free transitions (from S_0) are indicated. All energies are given with respect to the fully optimized singlet ground state energy.

			T_1	T_2	T_3	T_4	T_5	T_6	T_7	T_8	T_9	T_{10}
	f_{0i}	ΔE_{0i} / eV	2.48	2.65	2.82	2.98	3.06	3.17	3.19	3.26	3.27	3.32
S_0	-	0.27	0.6	0.8	4.1	1.0	0.9	0.4	0.9	0.4	0.2	2.0
S_1	0.0443	2.58	0.3	0.5	0.4	0.3	0.2	0.1	0.1	0.1	0.2	0.3
S_2	0.0134	2.88	0.3	0.4	0.2	0.5	0.3	0.1	0.2	0.1	0.0	0.2
S_3	0.0550	2.94	0.7	0.6	0.5	1.2	0.6	0.1	0.2	0.2	0.3	0.3
S_4	0.0355	3.05	0.4	0.8	0.6	0.2	0.6	0.1	0.2	0.1	0.1	0.3
S_5	0.0050	3.24	0.1	0.3	0.2	0.3	0.1	0.1	0.1	0.1	0.1	0.1
S_6	0.0014	3.25	0.2	0.3	0.2	0.1	0.2	0.1	0.2	0.1	0.1	0.1
S_7	0.0008	3.27	0.1	0.1	0.1	0.1	0.1	0.0	0.0	0.0	0.0	0.1
S_8	0.0130	3.30	0.3	0.3	0.1	0.2	0.5	0.1	0.0	0.0	0.1	0.3
S_9	0.0264	3.31	0.1	0.1	0.2	0.2	0.2	0.2	0.1	0.1	0.1	0.1
S_{10}	0.0734	3.44	0.1	0.3	0.2	0.1	0.5	0.1	0.0	0.1	0.1	0.2

Table S5: Rate constants for intersystem crossing (downhill ISC, k_{ISC} in s^{-1},), reverse ISC (uphill RISC, k_{RISC} in s^{-1}) as well as for the radiative component fluorescence and phosphorescence (k_F and k_P in s^{-1}) as calculated based on the scalar-relativistic TDDFT simulations. In case of considered structural relaxation k_{ISC} and k_{RISC} rates were obtained by means of $\langle T_1|\hat{H}_{SOC}|S_1\rangle$ within the S_1 equilibrium structure (0.1 cm^{-1}, upper value) and within the T_1 equilibrium structure (0.3 cm^{-1}, bottom value). Highlighted values are indicated in Figure 3 in the main manuscript.

	**Equilibrium Structure**	**$k_{ISC}(S_1 \rightarrow T_1)$ / s^{-1}**	**$k_{RISC}(T_1 \rightarrow S_1)$ / s^{-1}**	**$k_F(S_1 \rightarrow S_0)$ / s^{-1}**	**$k_P(T_1 \rightarrow S_0)$ / s^{-1}**
**Vertical**	S_0	1.12×10^7	3.40×10^3	-	-
	S_1	7.23×10^6	8.84×10^5	**3.12×10^6**	-
	T_1	1.85×10^7	3.24×10^5	-	**1.00**
**Structural relaxation**	S_1-T_1	8.23×10^6<br>**3.40×10^7**	1.96×10^6<br>**8.10×10^6**	-	-

5.2 Cobaloxime Catalyst

Table S6: Calculated electronic absorption spectrum of CAT cobaloxime as obtained at the PBE0/def2-svp level of theory in acetonitrile. Electronic properties of the lowest ten as well as dipole-allowed singlet-singlet are summarized, i.e., electronic characters, excitation energies, excitation wavelengths and oscillator strengths. Electronic characters are indicated by charge density difference plots, while charge transfer occurs from red to blue.

**Singlet-singlet**				
Transition $S_0 \rightarrow S_i$	Character	ΔE_{0i} / eV	λ / nm	f_{0i}
S_1	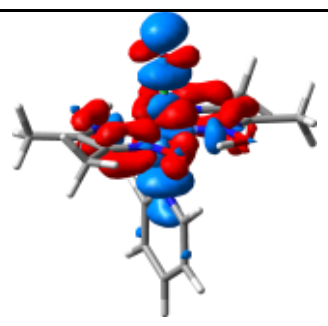	2.28	543	0.0000
S_2	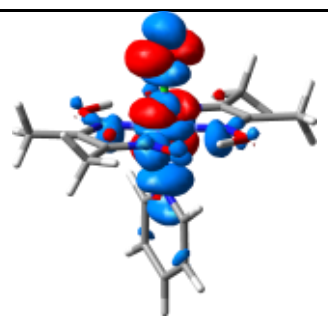	2.55	486	0.0001
S_3	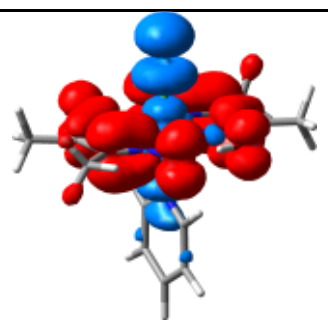	3.39	366	0.0000
S_4	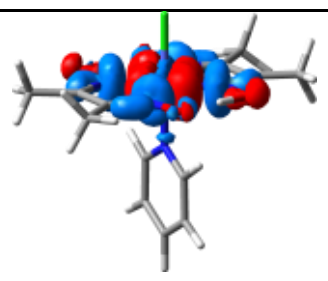	3.42	363	0.0001
S_5	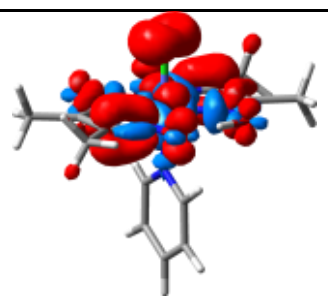	3.43	362	0.0001
S_6	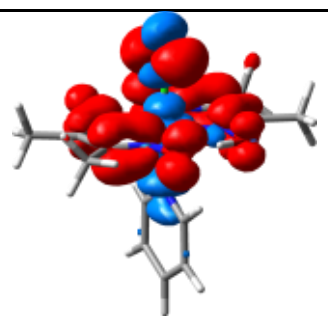	3.66	339	0.0004
S_7	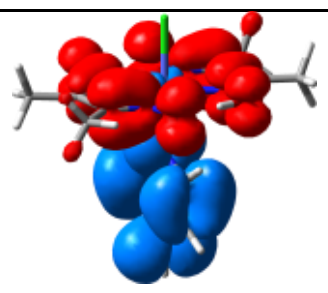	3.67	338	0.0000
S_8	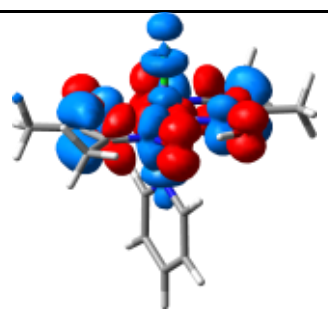	3.70	336	0.0003

S_9	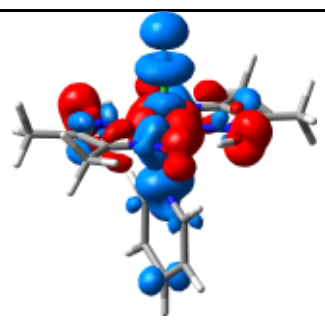	3.71	334	0.0000
S_{10}	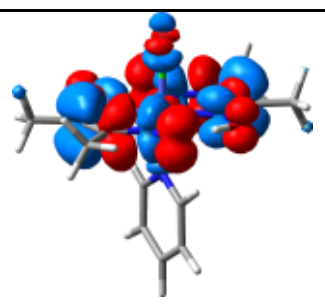	3.73	332	0.0246
S_{15}	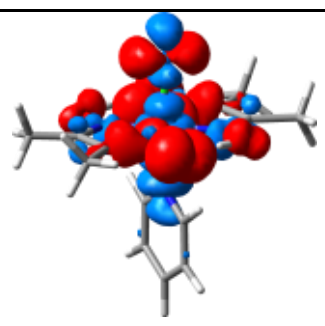	4.09	303	0.0414
S_{16}	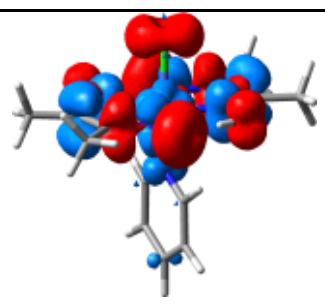	4.16	298	0.0229

Table S7: Energetic evaluation of intermediates involved in the first and second reduction of cobaloxime as obtained at the PBE0/def2-svp level of theory in acetonitrile. Relative energies (ΔE) are provided for the first reduction for doublet and quartet species as well as for the second reduction for singlet and triplet species. For each open-shell species (doublet, triplet, quartet) the spin density is given to illustrate the electronic character, while the highest occupied molecular orbital is shown for the closed-shell (singlet) species. Relative Gibbs free energies (ΔG) are provided respectively, but exclusively for the relaxed species.

**1. Reduction event**		Spin state, ΔE / eV		ΔG / eV
		Doublet	Quartet	
Equilibrium structure	D_0	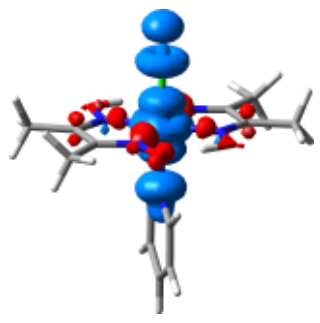	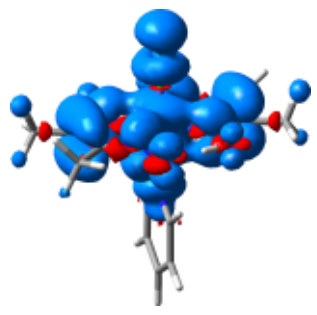	
	Q_0	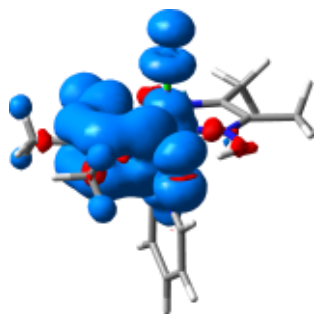	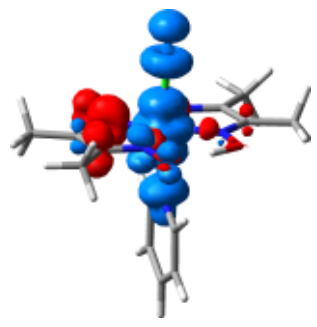	
**2. Reduction event**		Spin state, ΔE / eV		ΔG / eV
		Singlet	Triplet	
Equilibrium structure	S_0	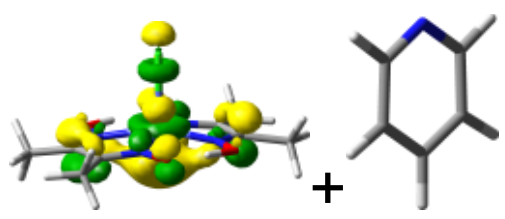	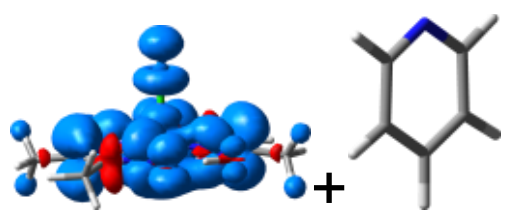	0.00
		0.27	0.90	
	T_1	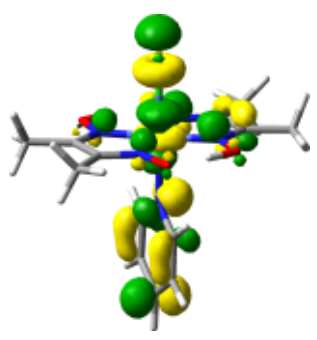	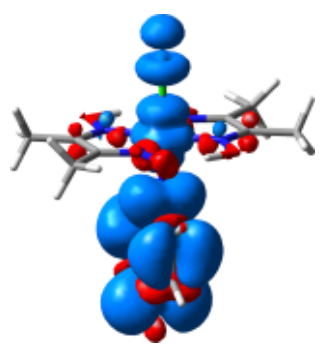	0.16
		1.50	0.00	

6 Radiometry

To determine the incident photon flux per vial, the photoreactor was characterized by spatially resolved two-dimensional (2D) radiometry as described by Sender et al.[14] The irradiation module was positioned at a distance of 8 cm from the detector, and the LEDs were operated at driving currents of 0.1, 0.35, 0.5, 0.7, and 1 A using a programmable DC power supply (Korad Lab KA3005P).

The scan was performed over an area of 60 cm × 60 cm using a pixel size of 0.45 cm, with integration times of 1.3–37 ms adjusted to the respective irradiance of the measurement. For evaluation, the map was cropped to the region of interest corresponding to the vial positions used in the experiments. The region was identified automatically as the bounding box of all pixels exceeding 50 % of the maximum irradiance.

The relative irradiance maps were converted into absolute irradiances (in W m^{-2}) using a correction factor derived from two direct reference measurements, with the LED module operated at 0.7 A and 1 A, respectively. Assuming that all emitted photons reach the detection plane, the integrated maps were normalized to the radiant-power values given in the manufacturer's datasheets (15.6 W at 0.7 A and 21.2 W at 1 A).[15,16]

The homogeneity of the irradiation is defined as $1 - \sigma/\mu$, where σ and μ are the standard deviation and the mean irradiance over the cropped area, respectively. The resulting current-dependent optical powers and homogeneities are summarized in Table S8. The incident optical power per vial was obtained by multiplying the mean irradiance by the projected cross-sectional area of a single vial (544 mm^2). At a driving current of 1 A, the mean irradiance of 73.4 W m^{-2} corresponds to an incident optical power of 39.9 mW per vial, which provides the basis for the photon-flux and apparent quantum yield calculation given in Section 4.1. The homogeneity of 0.82 indicates an approximately uniform irradiance distribution. The incident optical power is therefore assumed to be identical for all vials, independent of their position.

Table S8: Current-dependent mean irradiance, incident optical power per vial and irradiation homogeneity of the used irradiation module determined by 2D radiometry.

LED current / A	Mean irradiance / W m^{-2}	Incident optical power per vial / mW	Homogeneity / 1
0.10	10.1	5.5	0.825
0.35	32.3	17.6	0.823
0.50	44.0	23.9	0.823
0.70	57.4	31.2	0.822
1.00	73.4	39.9	0.832

7 Light-driven hydrogen evolution

All HER samples were prepared under an argon atmosphere. MeCN, MeOH, and H_2O were deoxygenated by purging with argon and were stored in the glovebox before use. Unless stated otherwise, the final reaction volume was 1.00 mL. Stock solutions were prepared as summarized in Table S9 and were combined according to Tables S10, S11 and S12. Additional MeCN, MeOH, or H_2O was added to reach the desired final solvent composition.

Dimethylglyoxime ($dmgH_2$) was added to the sacrificial electron donor stock solutions to stabilize the cobaloxime catalyst under photocatalytic conditions. Reduced cobaloxime intermediates are known to undergo ligand exchange and ligand dissociation, and the presence of free $dmgH_2$ can help maintain the active cobaloxime species during catalysis.[17,18] Samples were prepared in triplicate using a Gilson GX-241 Liquid Handler equipped with a Verity 4120 Dual Syringe Pump. After preparation, the GC vials were sealed and irradiated in a multi-batch screening photoreactor. The irradiation modules described by Kowalczyk et al. were modified with two 453 nm LEDs (OSRAM, LZ4-40B208-0000) mounted at the outermost heatsink positions with an LED distance of 200 mm.[19] In total, two LED modules with a total number of four LEDs were used. Hydrogen evolution was quantified by gas chromatography by injecting 100 µL of the vial headspace into a Shimadzu Nexis GC-2030 equipped with a SH-Msieve 5A column and a BID detector.

Table S9: Stock solutions used for the light-driven HER experiments.

Stock solution	Short name	Composition
Stock 1	AA/MeOH-H_2O	Ascorbic acid, 85 mg mL^{-1}, and dmgH_2, 5 mg mL^{-1}, in MeOH/H_2O 1:1 v/v. The solution was adjusted to pH 4 by addition of 25% aqueous NH_3 to form ammonium ascorbate.
Stock 2	AA/MeCN-H_2O	Ascorbic acid, 85 mg mL^{-1}, and dmgH_2, 5 mg mL^{-1}, in MeCN/H_2O 1:1 v/v. The solution was adjusted to pH 4 by addition of 25% aqueous NH_3 to form ammonium ascorbate.
Stock 3	TEA	TEA, 64 μL mL^{-1}, and dmgH_2, 5 mg mL^{-1}, in MeCN/H_2O 1:1 v/v.
Stock 4	TEOA	TEOA, 67 μL mL^{-1}, and dmgH_2, 5 mg mL^{-1}, in MeCN/H_2O 1:1 v/v.
Stock 5	4CzIPN/MeCN, 10 mM	10 mM 4CzIPN in MeCN.
Stock 6	4CzIPN/MeCN, 1 mM	1 mM 4CzIPN in MeCN.
Stock 7	4CzIPN/MeOH, 1 mM	1 mM 4CzIPN in MeOH.
Stock 8	Cobaloxime	0.1 mM ClPy[Co(dmgH)$_2$] in MeCN.
Stock 9	PDha-g-PEG	10 mg mL^{-1} PDha-g-PEG in MeCN/H_2O 1:1 v/v.

Table S10: Preparation of HER reaction mixtures for the solvent, 4CzIPN concentration, and PDha-g-PEG screening. The final reaction volume was 1.00 mL.

Component	Initial $MeOH/H_2O$ test	Initial $MeCN/H_2O$ test	1 mM 4CzIPN screening	0.1 mM 4CzIPN screening	0.1 mM 4CzIPN + PDha-g-PEG screening
Stock 1, AA/MeOH-H_2O	200 µL	200 µL	–	–	–
Stock 2, AA/MeCN-H_2O	–	–	200 µL	200 µL	200 µL
Stock 5, 10 mM 4CzIPN in MeCN	–	–	100 µL	–	–
Stock 6, 1 mM 4CzIPN in MeCN	–	–	–	100 µL	100 µL
Stock 7, 1 mM 4CzIPN in MeOH	100 µL	100 µL	–	–	–
Stock 8, 0.1 mM cobaloxime	20 µL	20 µL	20 µL	20 µL	20 µL
Stock 9, PDha-g-PEG	–	–	–	–	100 µL
Additional solvent	See Table S11	See Table S11	See Table S11	See Table S11	See Table S11

Table S11: Additional solvent volumes used to obtain the desired final solvent composition.

Screening series	Target solvent composition	Additional H_2O	Additional MeCN or MeOH
Initial MeOH/H_2O test	nominal MeOH/H_2O 4:1 v/v	100 µL H_2O	680 µL MeOH
Initial MeCN/H_2O test	nominal MeCN/H_2O 4:1 v/v	100 µL H_2O	680 µL MeCN
1 mM 4CzIPN screening, 15 vol.% H_2O	MeCN/H_2O, 15 vol.% H_2O	50 µL H_2O	630 µL MeCN
1 mM 4CzIPN screening, 20 vol.% H_2O	MeCN/H_2O, 20 vol.% H_2O	100 µL H_2O	580 µL MeCN
1 mM 4CzIPN screening, 30 vol.% H_2O	MeCN/H_2O, 30 vol.% H_2O	200 µL H_2O	480 µL MeCN
1 mM 4CzIPN screening, 40 vol.% H_2O	MeCN/H_2O, 40 vol.% H_2O	300 µL H_2O	380 µL MeCN
0.1 mM 4CzIPN screening, 15 vol.% H_2O	MeCN/H_2O, 15 vol.% H_2O	50 µL H_2O	630 µL MeCN
0.1 mM 4CzIPN screening, 20 vol.% H_2O	MeCN/H_2O, 20 vol.% H_2O	100 µL H_2O	580 µL MeCN
0.1 mM 4CzIPN screening, 30 vol.% H_2O	MeCN/H_2O, 30 vol.% H_2O	200 µL H_2O	480 µL MeCN
0.1 mM 4CzIPN screening, 40 vol.% H_2O	MeCN/H_2O, 40 vol.% H_2O	300 µL H_2O	380 µL MeCN

0.1 mM 4CzIPN + PDha-g-PEG, 15 vol.% H_2O	MeCN/H_2O, 15 vol.% H_2O	0 µL H_2O	580 µL MeCN
0.1 mM 4CzIPN + PDha-g-PEG, 20 vol.% H_2O	MeCN/H_2O, 20 vol.% H_2O	50 µL H_2O	530 µL MeCN
0.1 mM 4CzIPN + PDha-g-PEG, 30 vol.% H_2O	MeCN/H_2O, 30 vol.% H_2O	150 µL H_2O	430 µL MeCN
0.1 mM 4CzIPN + PDha-g-PEG, 40 vol.% H_2O	MeCN/H_2O, 40 vol.% H_2O	250 µL H_2O	330 µL MeCN

For the initial MeOH/H_2O test, the final solvent composition is described as nominal MeOH/H_2O 4:1 v/v; 20 µL MeCN was introduced through the cobaloxime stock solution. For all MeCN/H_2O screening experiments, the final concentrations were 2 µM cobaloxime, 0.1 M ammonium ascorbate, and either 1 mM or 0.1 mM 4CzIPN, as indicated. For the PDha-g-PEG series, the final polymer concentration was 1 mg mL^{-1}.

Table S12: Preparation of HER reaction mixtures for the catalyst concentration and SED screening. The final reaction volume was 1.00 mL.

Component	AA series	TEA series	TEOA series
Stock 2, AA/MeCN-H_2O	200 µL	–	–
Stock 3, TEA	–	200 µL	–
Stock 4, TEOA	–	–	200 µL
Stock 6, 1 mM 4CzIPN in MeCN	100 µL	100 µL	100 µL
Stock 8, 0.1 mM cobaloxime	10, 20, 40, or 60 µL	20, 40, or 60 µL	10, 20, 40, or 60 µL
Additional H_2O	100 µL	100 µL	100 µL
Additional MeCN	adjusted to 1.00 mL	adjusted to 1.00 mL	adjusted to 1.00 mL

The volume of Stock 8 was varied to obtain final cobaloxime concentrations of 1, 2, 4, or 6 μM. Specifically, 10, 20, 40, and 60 μL of the 0.1 mM cobaloxime stock solution correspond to final cobaloxime concentrations of 1, 2, 4, and 6 μM, respectively, in a final volume of 1.00 mL. For all catalyst concentration and SED screening experiments, the final solvent composition was MeCN/H_2O containing 20 vol.% H_2O, and the final 4CzIPN concentration was 0.1 mM.

For clarity, the corresponding additional MeCN volumes were 590, 580, 560, and 540 μL for final cobaloxime concentrations of 1, 2, 4, and 6 μM, respectively

Table S13: Influence of cobaloxime concentration and SED on light-driven HER with 4CzIPN as photosensitizer. TONs were determined after 20 h irradiation at 453 nm. General conditions: 0.1 mM 4CzIPN, 1 mg/mL dmgH$_2$, and 0.1 M sacrificial electron donor in MeCN/H$_2$O containing 20 vol.% H$_2$O. TON values were calculated with respect to the catalyst and are reported as mean ± standard deviation from triplicate measurements.

Cobaloxime concentration [µM]	Ammonium ascorbate	TEA	TEOA
1	6451 ± 82	6.4 ± 0.7	190 ± 4
2	5030 ± 166	119 ± 20	161 ± 6
4	2688 ± 189	405 ± 32	246 ± 5
6	2001 ± 66	790 ± 69	281 ± 7

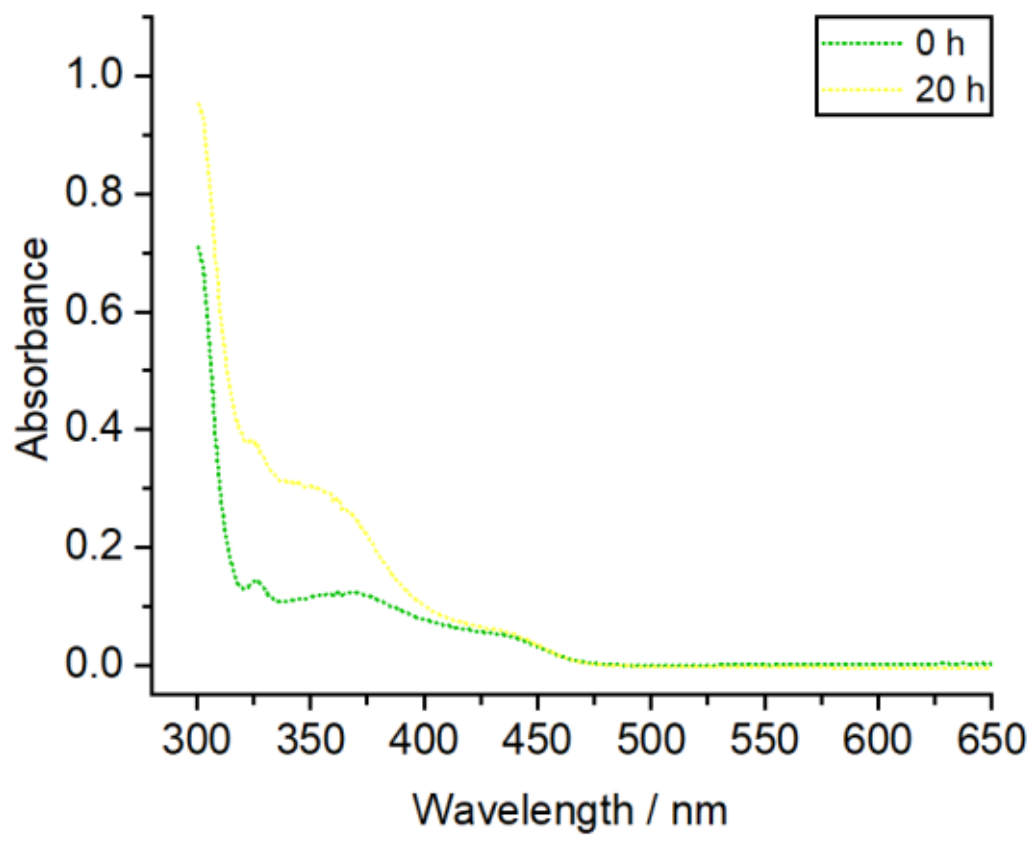

Figure S4: UV/Vis spectra of the HER reaction mixture before irradiation and after 20 h of light-driven hydrogen evolution under optimized conditions: 0.1 mM 4CzIPN, 1 µM cobaloxime, 1 mg/mL dmgH$_2$, and 0.1 M ammonium ascorbate in MeCN/H$_2$O containing 20 vol.% H$_2$O; irradiation at 453 nm. Spectra were recorded directly in the GC vials using a 3D-printed UV/Vis cell equipped with COL-UV/Vis collimating lenses with 3/8”-24 nuts. The cell was connected via optical fibers (FC-UVIR600-1-BX) to an AvaLight-DH-S-BAL light source and an AvaSpec-VRS2048CL-EVO spectrometer.[19]

Table S14: Literature comparison of selected cobaloxime- or cobalt-based light-driven HER systems with different photosensitizers. TON values* are reported as given in the original publications and are shown with respect to the catalyst and/or photosensitizer where available. Direct comparison should be made with caution because the systems differ in solvent composition, sacrificial electron donor, irradiation time, catalyst loading, photosensitizer loading, and TON calculation method.

Catalyst	PS	TON (cat.)	TON (PS)	Ref.
ClPy[Co(dmgH)$_2$]	DAHA	78 (160 h)	78,000 (160 h)	[20]
ClPy[Co(dmgH)$_2$]	4CzIPN	6,451 (20 h)	64 (20 h)	this work
ClPy[Co(dmgH)$_2$]	[Pt(ttpy)(C≡CPh)]ClO$_4$	2,150 (10 h)	21,500 (10 h)	[21]
ClPy[Co(dmgH)$_2$]	Et$_2$N-KC-(OMe)$_3$	3,820 (20 h)	3.8 (20 h)	[22]
PdCl$_2$(PPh$_3$)$_2$	4CzIPN	1767 (60 h)	2,020 (60 h)	[23]
[Co(dmgH)$_2$(py)2]$^+$ BArF$^-$	[Ru(bpy)$_3$](PF$_6$)$_2$	65 (24 h)	65 (24 h)	[24]
Im-EtPMO-Co	eosin Y	138 (2.5 h)	-	[25]
py-etPMO–Co	eosin Y	113 (4 h)	-	[26]

*TON(cat.) and TON(PS) refer to turnover numbers calculated with respect to the amount of catalyst and photosensitizer, respectively. Empty entries indicate that the corresponding value was not reported or could not be unambiguously extracted from the original publication

8 AQY Calculation

The apparent quantum yield (AQY) was calculated using the following equation:

$$\phi = \frac{nR}{I}$$

Where ϕ is the AQY, n the number of electrons required per molecule of hydrogen, R the amount of hydrogen produced, and I the number of incident photons. For hydrogen evolution the value n was two, since two electrons are required per molecule of H_2. Radiometric measurements of the system yielded a mean irradiance of 73.4 W m^{-2} at an applied current of 1 A and an LED peak emission wavelength of 453 nm. With a projected cross-sectional area of 544 mm^2 per vial, the incident optical power per vial was 39.9 mW. Using a monochromatic approximation for the LED at its peak emission wavelength, a corresponding photon flux of 151 nmol/s was determined. Over an irradiation period of 20 h (72,000 s), the total number of incident photons was therefore 10.89 mmol, resulting in a total AQY of ϕ=0.123 %, for the best conditions identified in the screening, using 0.1 mM 4CzIPN, 1 μM cobaloxime, 1 mg/mL $dmgH_2$, and 0.1 M ammonium ascorbate in MeCN/H_2O containing 20 vol.% H_2O.

9 Stern-Volmer Quenching Experiments

For the Stern-Volmer quenching experiments two solutions were prepared under anaerobic conditions in two cuvettes with septa. Solution one (S1) contained 4CzIPN in a concentration of 0.18 µM (acetonitrile/water, 1/1 (v/v)), and solution two (S2) contained 4CzIPN (0.18 µM, acetonitrile/water, 1/1 (v/v)) and ascorbic acid (0.24 M, acetonitrile/water, 1/1 (v/v)). In addition, 15 µL of an aqueous ammonia solution was transferred to solution S2 to form the ammonium ascorbate.

From solution S1, emission decays of prompt (0 – 400 ns) and delayed fluorescence (0 – 13 µs) were measured by TCSPC spectroscopy. This way the emission lifetime of the prompt and delayed fluorescence without quencher was determined. In the following a certain volume of solution S2 was transferred into cuvette one with a HAMILTON syringe and emission lifetime was measured. This procedure was repeated several times to obtain the emission lifetime with increasing amount of quencher for the Stern-Volmer plots. The values obtained in this way are displayed in Tables S15 and S15.

Table S15: Emission lifetime (prompt fluorescence) of 4CzIPN with an increasing amount of quencher (ammonium ascorbate) added.

Name	Conc. ammonium ascorbate / mM	τ_{PF} / ns
Solution 1 (S1)	0.0	6.2
S1 + 10 µL S2	1.2	5.2
S1 + 20 µL S2	2.4	5.3
S1 + 30 µL S2	3.5	5.1
S1 + 40 µL S2	4.7	4.9
S1 + 50 µL S2	5.8	4.7
S1 + 100 µL S2	11.4	4.1
S1 + 200 µL S2	21.8	3.2
S1 + 400 µL S2	39.9	2.2

Table S16: Emission lifetime (delayed fluorescence) of 4CzIPN with an increasing amount of quencher (ammonium ascorbate) added.

Name	Conc. Ammonium Ascorbate / mM	τ_{DF} / ns
Solution 1	0.0	1522
S1 + 10 µL S2	0.6	1245.1
S1 + 20 µL S2	1.2	1200
S1 + 30 µL S2	1.8	1073.8
S1 + 40 µL S2	2.4	992.2
S1 + 50 µL S2	2.9	920.4
S1 + 60 µL S2	3.5	872.7
S1 + 70 µL S2	4.1	865.2
S1 + 80 µL S2	4.7	839.3
S1 + 90 µL S2	5.2	799.7
S1 + 100 µL S2	5.8	793.1
S1 + 150 µL S2	8.4	613.5

10 References

[1] M. Krejčik, M. Daněk, F. Hartl, “Simple construction of an infrared optically transparent thin-layer electrochemical cell” *Journal of Electroanalytical Chemistry and Interfacial Electrochemistry* **1991**, *317*, 179–187.

[2] E. Speckmeier, T. G. Fischer, K. Zeitler, “A Toolbox Approach To Construct Broadly Applicable Metal-Free Catalysts for Photoredox Chemistry: Deliberate Tuning of Redox Potentials and Importance of Halogens in Donor–Acceptor Cyanoarenes” *J. Am. Chem. Soc.* **2018**, *140*, 15353–15365.

[3] M. J. Frisch, G. W. Trucks, H. B. Schlegel, G. E. Scuseria, M. A. Robb, J. R. Cheeseman, G. Scalmani, V. Barone, G. A. Petersson, H. Nakatsuji, X. Li, M. Caricato, A. V. Marenich, J. Bloino, B. G. Janesko, R. Gomperts, B. Mennucci, H. P. Hratchian, J. V. Ortiz, A. F. Izmaylov, J. L. Sonnenberg, D. Williams-Young, F. Ding, F. Lipparini, F. Egidi, J. Goings, B. Peng, A. Petrone, T. Henderson, D. Ranasinghe, V. G. Zakrzewski, J. Gao, N. Rega, G. Zheng, W. Liang, M. Hada, M. Ehara, K. Toyota, R. Fukuda, J. Hasegawa, M. Ishida, T. Nakajima, Y. Honda, O. Kitao, H. Nakai, T. Vreven, K. Throssell, J. A. Montgomery, Jr., J. E. Peralta, F. Ogliaro, M. J. Bearpark, J. J. Heyd, E. N. Brothers, K. N. Kudin, V. N. Staroverov, T. A. Keith, R. Kobayashi, J. Normand, K. Raghavachari, A. P. Rendell, J. C. Burant, S. S. Iyengar, J. Tomasi, M. Cossi, J. M. Millam, M. Klene, C. Adamo, R. Cammi, J. W. Ochterski, R. L. Martin, K. Morokuma, O. Farkas, J. B. Foresman, D. J. Fox **2016**.

[4] C. Adamo, V. Barone, “Toward reliable density functional methods without adjustable parameters: The PBE0 model” *The Journal of Chemical Physics* **1999**, *110*, 6158–6170.

[5] F. Weigend, R. Ahlrichs, “Balanced basis sets of split valence, triple zeta valence and quadruple zeta valence quality for H to Rn: Design and assessment of accuracy” *Phys. Chem. Chem. Phys.* **2005**, *7*, 3297.

[6] G. D. Scholes, C. Curutchet, B. Mennucci, R. Cammi, J. Tomasi, “How Solvent Controls Electronic Energy Transfer and Light Harvesting” *J. Phys. Chem. B* **2007**, *111*, 6978–6982.

[7] A. V. Marenich, C. J. Cramer, D. G. Truhlar, “Universal Solvation Model Based on Solute Electron Density and on a Continuum Model of the Solvent Defined by the Bulk Dielectric Constant and Atomic Surface Tensions” *J. Phys. Chem. B* **2009**, *113*, 6378–6396.

[8] S. Grimme, S. Ehrlich, L. Goerigk, “Effect of the damping function in dispersion corrected density functional theory” *Journal of Computational Chemistry* **2011**, *32*, 1456–1465.

[9] F. Neese *Wiley Interdiscip. Rev. Comput. Mol. Sci.* **2012**, 73–78.

[10] D. A. Pantazis, F. Neese, “All-electron scalar relativistic basis sets for the 6p elements” *Theor Chem Acc* **2012**, *131*, 1292.

[11] K. Shizu, H. Kaji, “Theoretical Determination of Rate Constants from Excited States: Application to Benzophenone” *J. Phys. Chem. A* **2021**, *125*, 9000–9010.

[12] D. P. Craig, T. Thirunamachandran, *Molecular Quantum Electrodynamics: An Introduction to Radiation-molecule Interactions*, Academic Press Inc, **1998**.

[13] R. A. Arellano-Reyes, A. Prabhakaran, R. C. E. Sia, J. Guthmuller, K. K. Jha, T. Yang, B. Dietzek-Ivanšić, V. McKee, T. E. Keyes, “BODIPY-Perylene Charge Transfer Compounds; Sensitizers for Triplet-Triplet Annihilation Up-conversion” *Chemistry – A European Journal* **2023**, *29*, e202300239.

[14] M. Sender, B. Wriedt, D. Ziegenbalg, “Radiometric measurement techniques for in-depth characterization of photoreactors – part 1: 2 dimensional radiometry” *React. Chem. Eng.* **2021**, *6*, 1601–1613.

[15] OSRAM LZ4-40B208 Datasheet, https://look.ams-osram.com/m/4f9578412571e162/original/LZ4-40B208.pdf **19.06.2026**.
[16] OSRAM LZ4-00B208 Datasheet, https://look.ams-osram.com/m/38caeaf74b4bbbb3/original/LZ4-00B208.pdf **19.06.2026**.
[17] R. Dong, K.-K. Chen, P. Wang, N. Zhang, S. Guo, Z.-M. Zhang, T.-B. Lu, "Heavy atom-free Keto-di-coumarin as earth-abundant strong visible light-harvesting photosensitizer for efficient photocatalytic hydrogen evolution" *Dyes and Pigments* **2019**, *166*, 84–91.
[18] J. L. Dempsey, B. S. Brunschwig, J. R. Winkler, H. B. Gray, "Hydrogen Evolution Catalyzed by Cobaloximes" *Acc. Chem. Res.* **2009**, *42*, 1995–2004.
[19] D. Kowalczyk, G. Knorr, K. Peneva, D. Ziegenbalg, "Making photocatalysts screenable – a milliscale multi-batch screening photoreactor as extension for the modular photoreactor" *React. Chem. Eng.* **2023**, *8*, 2967–2983.
[20] M. Ming, H. Yuan, S. Yang, Z. Wei, Q. Lei, J. Lei, Z. Han, "Efficient Red-Light-Driven Hydrogen Evolution with an Anthraquinone Organic Dye" *J. Am. Chem. Soc.* **2022**, *144*, 19680–19684.
[21] P. Du, J. Schneider, G. Luo, W. W. Brennessel, R. Eisenberg, "Visible Light-Driven Hydrogen Production from Aqueous Protons Catalyzed by Molecular Cobaloxime Catalysts" *Inorg. Chem.* **2009**, *48*, 4952–4962.
[22] G. Knorr, K. Hotzel, A. Chettri, A. Skabeev, M. Wächtler, B. Dietzek-Ivanšić, K. Peneva, "Unlocking the potential of ketocoumarins: efficient photosensitizers for sustainable light driven hydrogen evolution" *J. Mater. Chem. A* **2023**, *11*, 23260–23269.
[23] Z.-J. Yu, W.-Y. Lou, H. Junge, A. Päpcke, H. Chen, L.-M. Xia, B. Xu, M.-M. Wang, X.-J. Wang, Q.-A. Wu, B.-Y. Lou, S. Lochbrunner, M. Beller, S.-P. Luo, "Thermally activated delayed fluorescence (TADF) dyes as efficient organic photosensitizers for photocatalytic water reduction" *Catalysis Communications* **2019**, *119*, 11–15.
[24] E. Oswald, A.-L. Gaus, J. Kund, M. Küllmer, J. Romer, S. Weizenegger, T. Ullrich, A. K. Mengele, L. Petermann, R. Leiter, P. R. Unwin, U. Kaiser, S. Rau, A. Kahnt, A. Turchanin, M. von Delius, C. Kranz, "Cobaloxime Complex Salts: Synthesis, Patterning on Carbon Nanomembranes and Heterogeneous Hydrogen Evolution Studies" *Chemistry – A European Journal* **2021**, *27*, 16896–16903.
[25] M. Á. Navarro, M. A. Martín, J. R. Ruiz, C. Jiménez-Sanchidrián, F. J. Romero-Salguero, D. Esquivel, "Improved Photocatalytic H2 Evolution by Cobaloxime-Tethered Imidazole-Functionalized Periodic Mesoporous Organosilica" *Hydrogen* **2023**, *4*, 120–132.
[26] M. Á. Navarro, D. Cosano, A. Bhunia, L. Simonelli, V. Martin-Diaconescu, F. J. Romero-Salguero, D. Esquivel, "Cobaloxime tethered pyridine-functionalized ethylene-bridged periodic mesoporous organosilica as an efficient HER catalyst" *Sustainable Energy Fuels* **2022**, *6*, 398–407.
